# YhdP, TamB, and YdbH Are Redundant but Essential for Growth and Lipid Homeostasis of the Gram-Negative Outer Membrane

**DOI:** 10.1128/mBio.02714-21

**Published:** 2021-11-16

**Authors:** Natividad Ruiz, Rebecca M. Davis, Sujeet Kumar

**Affiliations:** a Department of Microbiology, The Ohio State University, Columbus, Ohio, USA; National Cancer Institute

**Keywords:** AsmA-like proteins, phospholipid transport, outer membrane biogenesis, envelope biogenesis, synthetic lethality

## Abstract

The bacterial cell envelope is the first line of defense and point of contact with the environment and other organisms. Envelope biogenesis is therefore crucial for the survival and physiology of bacteria and is often targeted by antimicrobials. Gram-negative bacteria have a multilayered envelope delimited by an inner and outer membrane (IM and OM, respectively). The OM is a barrier against many antimicrobials because of its asymmetric lipid structure, with phospholipids composing the inner leaflet and lipopolysaccharides (LPS) the outer leaflet. Since lipid synthesis occurs at the IM, phospholipids and LPS are transported across the cell envelope and asymmetrically assembled at the OM during growth. How phospholipids are transported to the OM remains unknown. Recently, the Escherichia coli protein YhdP has been proposed to participate in this process through an unknown mechanism. YhdP belongs to the AsmA-like clan and contains domains homologous to those found in lipid transporters. Here, we used genetics to investigate the six members of the AsmA-like clan of proteins in E. coli. Our data show that YhdP and its paralogs TamB and YdbH are redundant, but not equivalent, in performing an essential function in the cell envelope. Among the AsmA-like paralogs, only the combined loss of YhdP, TamB, and YdbH is lethal, and any of these three proteins is sufficient for growth. We also show that these proteins are required for OM lipid homeostasis and propose that they are the long-sought-after phospholipid transporters that are required for OM biogenesis.

## INTRODUCTION

**FIG 1:**
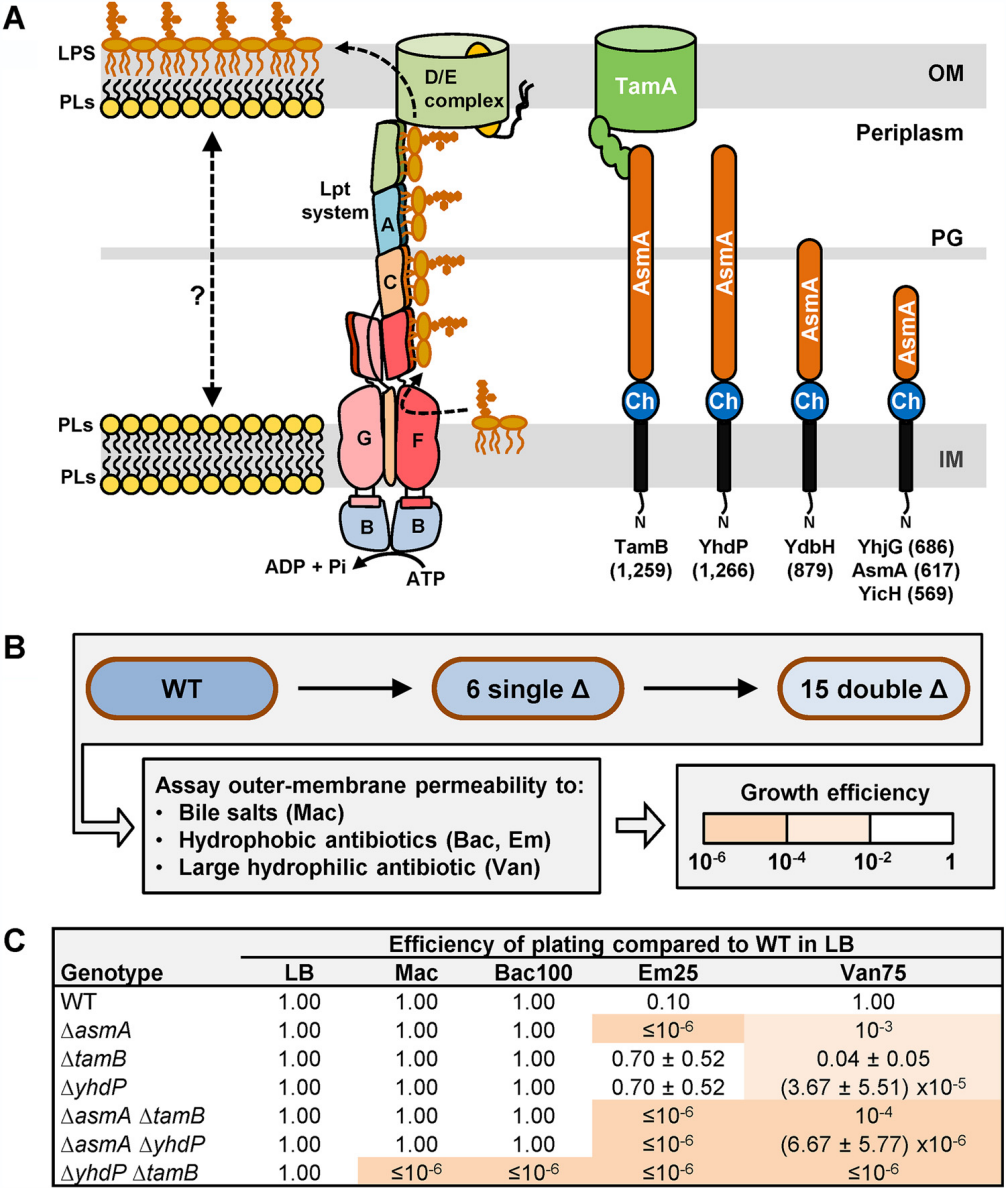
TamB and YhdP are functionally redundant for envelope integrity. (A) Cartoon representation of the E. coli cell envelope showing the IM and OM, periplasm, and peptidoglycan (PG) layer. On the left, the localization and transport of the major lipid components of the IM and OM are shown. Newly synthesized LPS is transported unidirectionally (dotted arrow) to the outer leaflet of the OM by the Lpt system (LptA-G proteins, labeled A to G), while phospholipids (PLs) are transported bidirectionally (dotted double arrow) between the IM and OM. On the right, cellular localization and predicted domain organization of the six AsmA-like proteins in E. coli are shown. Each AsmA-like protein (labeled at the bottom with number of amino acids shown in parentheses) is predicted to be anchored to the IM by an N-terminal transmembrane helix of ca. 22 aa. In the periplasm, they contain a region of ca. 120 aa (Ch) that is homologous to the chorein-N domain found in eukaryotic proteins involved in intermembrane (interorganelle) lipid transporters and a large region varying in length (ca. 425 to 1,125 aa) that is predicted to contain several AsmA domains. TamB has been shown to physically interact with the OM β-barrel protein TamA. (B) Strategy to construct and characterize the 6 mutants lacking one AsmA-like factor (single Δ) and the 15 mutants lacking two of these factors (double Δ). The quality of the barrier function of the OM was assessed by growing all strains in the presence of various antibiotics and bile salts and comparing their growth to that of the wild-type (WT) MG1655 strain in the absence of these compounds (see Materials and Methods for details). (C) Only the Δ*asmA*, Δ*yhdP*, and Δ*tamB* single mutants showed increased OM permeability (see Table S1A). All combinations of two alleles behaved in additive fashion except in the Δ*yhdP* Δ*tamB* double mutant, which exhibited synthetic defective phenotypes (see also Table S1A). Mac refers to MacConkey (bile salts), Bac100, 100 μg/ml bacitracin; Em25, 25 μg/ml erythromycin; Van75, 75 μg/ml vancomycin. Data represent the average and standard deviation from three biological replicates. If not shown, standard deviation equals zero.

The cell envelope of Gram-negative bacteria contains two essential membranes of distinct composition and function that are separated by an aqueous periplasmic compartment (1, 2). The inner membrane (IM) is a phospholipid bilayer that surrounds the cytoplasm. The outer membrane (OM), which separates the periplasm and the environment, has an inner leaflet composed of phospholipids and an outer leaflet composed of lipopolysaccharides (LPS) (3) (Fig. 1A). The structure and packing of LPS molecules at the cell surface limit the passage of hydrophobic molecules that would otherwise diffuse across phospholipids bilayers. Therefore, the innate resistance of Gram-negative bacteria to hydrophobic antibiotics and detergents depends on the asymmetric assembly of lipids at the OM (4, 5).

During growth, Gram-negative bacteria must expand the IM and OM in coordinated fashion. Since OM lipids are made at the IM (6, 7), growth requires the transport of newly synthesized phospholipids and LPS across the cell envelope (Fig. 1A). Decades ago, Osborn and colleagues demonstrated that, in Salmonella, LPS is transported from the IM to the OM, while phospholipids flow bidirectionally between these bilayers (8, 9). Since then, we have learned that LPS is transported across the cell envelope by the Lpt system, a multiprotein complex bridging the IM and OM (1, 10) (Fig. 1A). Lpt functions unidirectionally, as it is powered by an ATP-dependent transporter that moves LPS molecules from the outer leaflet of the IM to the periplasmic Lpt components (11); in addition, LPS translocation across the OM by the LptDE translocon requires activation through a periplasmic domain (12). In contrast, the mechanism for the bidirectional transport of phospholipids remains the most poorly understood essential biogenesis process in the Gram-negative cell envelope. Two types of transport could account for the bidirectional flow of phospholipids between the IM and OM. First, transport could be mediated by protein-based systems spanning the cell envelope (13). Recently, LetB and PqiB have been shown to assemble into tunnel-like structures that have been proposed to bridge the IM and OM and transport phospholipids (14–16). However, their function in cells remains unknown (1, 17). Alternatively, transport could involve hemifusion structures formed by fusion of the outer leaflet of the IM and the inner leaflet of the OM into a contiguous bilayer crossing the periplasm (13). Proteins could still be involved in the latter model by mediating the formation of hemifusion sites and/or cargo selectivity.

Recently, single-cell imaging has shown that IM-to-OM anterograde phospholipid transport occurs by diffusive flow in an Escherichia coli mlaA* mutant, in which phospholipids are mislocalized from the inner to the outer leaflet of the OM (18, 19). In the Mla system, wild-type MlaA is proposed to remove phospholipids that are somehow mislocalized to the cell surface and transfer them to the periplasmic protein MlaC, which transports them to the MlaBDEF complex to be inserted in the IM in an ATP-dependent manner (20–22). In contrast, MlaA* appears to work in reverse, aberrantly delivering phospholipids to the outer leaflet of the OM (19). In addition, when phospholipids are mislocalized to the cell surface, they can activate the OM phospholipase PldA, which attempts to restore OM lipid asymmetry in two ways: by breaking down the mislocalized phospholipids and by increasing LPS synthesis, likely at the expense of decreasing phospholipid synthesis, through a regulatory cascade that is triggered when the fatty acids released by PldA are recycled into the cytoplasm (23). This strategy contributes to OM lipid homeostasis in wild-type cells. However, when combined with the constitutive mistranslocation of phospholipids to the outer leaflet of the OM in mlaA* cells, it destabilizes the OM and leads to loss of OM material through blebbing, which further drives the IM-to-OM flow of phospholipids (19). The mlaA* cells tolerate this increased IM-to-OM flow during exponential growth because lipid synthesis is high; however, when lipid synthesis decreases in stationary phase owing to nutrient limitation, the sustained high demand of IM-to-OM phospholipid transport causes the IM to shrink more and eventually rupture, causing lysis of mlaA* cells (19). Importantly, a genetic approach enriching for mutations that slow down this anterograde phospholipid transport (i.e., lysis) in mlaA* cells identified loss-of-function mutations in *yhdP* (18). The rate of lysis of mlaA* *yhdP* cells was shown to be slower than that in the mlaA* parent because of slower IM shrinking, implicating YhdP in IM-to-OM anterograde phospholipid transport.

The function of YhdP is poorly understood. It was first identified as being required for SDS resistance in carbon-limited E. coli (24). In addition, the loss of YhdP causes mild OM permeability defects that can be suppressed by eliminating the synthesis of the cyclic form of enterobacterial common antigen (ECA_CYC_), a periplasmic glycan of unknown function (25). However, the effect of YhdP on anterograde phospholipid transport in mlaA* cells is independent of ECA_CYC_ (18). How YhdP modulates the effect of ECA_CYC_ on OM permeability and phospholipid transport in mlaA* strains remains unknown, as is whether YhdP affects phospholipid transport in wild-type cells.

It is logical to assume that anterograde phospholipid transport to the OM is essential for growth, since phospholipids are the lipid constituent of the inner leaflet of the OM and the OM is essential for survival. However, YhdP is not essential (24). In addition, the loss of YhdP slows down but does not abolish anterograde phospholipid transport in mlaA* cells (18). One interpretation of these facts is that YhdP only plays an accessory role in anterograde phospholipid transport. However, its predicted architecture suggests that YhdP directly transports phospholipids. YhdP is a 1,266-residue protein that belongs to the AsmA-like CL0401 protein clan (26). AsmA-like proteins contain a predicted IM-anchoring N-terminal α-helix and a large periplasmic domain that includes a chorein-N domain and the AsmA-like domain (Fig. 1A) (27, 28). Chorein-N domains are present in eukaryotic intermembrane lipid transporters, while AsmA-like domains are composed of various numbers of β-taco domains whose structure resembles that of the domains forming the periplasmic Lpt bridge, which shields the fatty acyl chains of LPS molecules as they travel through the periplasm en route to the OM (Fig. 1A) (29, 30). If YhdP indeed transported phospholipids, it would have to be functionally redundant with other transporters, since it is not essential for viability. Interestingly, E. coli encodes YhdP and five additional AsmA-like proteins (TamB [formerly YtfN], YdbH, AsmA, YicH, and YhjG [Fig. 1; see also Fig. S1 in the supplemental material]), and functional redundancy has been suggested as an explanation for why factors involved in IM-to-OM phospholipid transport have remained elusive (1, 31). These connections motivated the present study investigating the possible functional redundancy among AsmA-like proteins in E. coli. Using a genetic approach, we found that TamB, YhdP, and YdbH are redundant in performing a function that is essential in envelope biogenesis. We show that the combined loss of TamB, YhdP, and YdbH is lethal, and that mutants lacking five AsmA-like factors grow if they retain either TamB, YhdP, or YdbH. Based on our data and the fact that these proteins are predicted to have domains and overall structures similar to those present in lipid transporters, we propose that TamB, YhdP, and YdbH are the long-sought-after transporters of phospholipids between the IM and OM.

10.1128/mBio.02714-21.1 FIG S1 N-terminal periplasmic region of YhdP and AsmA-like paralogs is homologous to chorein-N domains present in eukaryotic lipid transporters. Hit list produced by searching residues 30 to 180 of YhdP with HHpred (supplemental material text reference 8) showing that the N-terminal periplasmic region of the six E. coli AsmA-like proteins (in blue) share sequence homology with each other and the N terminus of members of the eukaryotic chorein-N family of lipid transporters Atg2 and Vps13 (in orange), which are involved in interorganelle lipid transport (supplemental material text references 9–12). Mdm31 and Mdm32 (in green) are also eukaryotic proteins that have been implicated in intermembrane transport between the mitochondrial inner and outer membranes (supplemental material text reference 13). The sequence [referred to as YhdP(30-180)] corresponding to the first 151 residues (positions 30 to 180) of the predicted periplasmic region of YhdP was used to search for sequence homology in the PDB_mmCIF30_13_Sept and NCBI_Conserved_Domains(CD)_v3.18 databases. The top 11 of 15 hits are shown (hits 12 to 15 had <50% probability score). Prob refers to the probability of being a true positive hit. The E‐value is the average number of false positives with a score better than the one obtained for YhdP(30-180), while the *P* value is the E‐value divided by the number of sequences in the database. (B) Cartoon diagram of the crystal structure (PDB entry 6CBC) of residues 6 to 320 of the fungal *Chaetomium thermophilum* Vps13 protein containing the chorein-N domain. HHpred predicts homology with AsmA-like proteins up to ca. residue 190 (labeled). (C) Cartoon diagram of the crystal structure (PDB entry 5VTG) of the β-taco fold composed of residues 977 to 1136 of TamB from E. coli. Crystal structures are colored from blue (N terminus) to red (C terminus). Structure pictures were generated using PyMOL Molecular Graphics System (Schrödinger, LLC). (D) Cartoon representation of the model structures for Atg2 (UniProt entry P53855), YhdP (UniProt entry P46474), TamB (UniProt entry P39321), and YdbH (UniProt entry P52645) generated by AlfaFold (7). The Atg2 chain is colored using the rainbow pattern from blue (N terminus) to red (C terminus). In YhdP, TamB, and YdbH, the predicted transmembrane helix is in black, the chorein-N domain in blue, and the AsmA-like domain in orange. Download FIG S1, PDF file, 0.8 MB. Copyright © 2021 Ruiz et al. 2021 Ruiz et al. https://creativecommons.org/licenses/by/4.0/ This content is distributed under the terms of the Creative Commons Attribution 4.0 International license.

## RESULTS

### TamB and YhdP are functionally redundant in maintaining the barrier function of the OM.

The six paralogs of AsmA-like proteins in E. coli contain a chorein-N domain that is present in some intermembrane eukaryotic transporters (see Fig. S1 in the supplemental material). In addition, the recently released AlphaFold Protein Structure Database predicts that they share an overall structure with Atg2, a protein that belongs to a new family of lipid transporters at membrane-contact sites between eukaryotic organelles (Fig. S1) (32–35). Based on these relationships and the fact that YhdP has been shown to affect phospholipid transport from the IM to the OM (18), we investigated a possible role for the AsmA-like protein family in OM biogenesis in E. coli. We generated mutants lacking one or more of the six paralogs and tested for defects in growth and OM permeability, as defects in OM structure affects these phenotypes (2, 4). We first characterized the six single mutants. None of them exhibited growth defects in lysogeny broth (LB) at 37°C. To probe OM permeability, we monitored their resistance to bile salts and various antibiotics whose entry is limited by the OM (36). We did not detect OM permeability defects in the Δ*ydbH*, Δ*yhjG*, and Δ*yicH* single mutants, but, as previously reported in various Gram-negative bacteria, the Δ*asmA*, Δ*tamB*, and Δ*yhdP* mutants showed a slight increase in OM permeability (Fig. 1B and C, Table S1A) (24, 25, 37–39).

10.1128/mBio.02714-21.6 TABLE S1 Efficiency of plating data to assay OM permeability in mutants lacking one or two AsmA-like proteins (A), *tamA* (B), *mlaA* and *pldA* (C), or *wecA* (D). Download Table S1, XLSX file, 0.02 MB. Copyright © 2021 Ruiz et al. 2021 Ruiz et al. https://creativecommons.org/licenses/by/4.0/ This content is distributed under the terms of the Creative Commons Attribution 4.0 International license.

Next, we constructed mutants carrying multiple deletion alleles to determine if AsmA-like proteins are functionally redundant or function in different or the same pathways. We expected that loss of redundant factors would result in a phenotype greater than the one expected from adding the phenotype of the single mutants. In contrast, if the effect of combining deletions was additive, it would indicate that the respective factors function in independent pathways. A lack of phenotypic synergy or additivity would be expected if factors functioned in the same pathway. Lastly, a mutant allele might suppress phenotypes conferred by other alleles, revealing that the factors are somehow functionally related. We therefore constructed the 15 double mutants lacking AsmA-like proteins and analyzed the barrier function of their OM (Fig. 1B). We observed that adding either Δ*ydbH*, Δ*yhjG*, or Δ*yicH* to any single mutant did not alter their respective OM permeability phenotypes (Table S1A). Since, on their own, these three alleles do not confer any phenotypes, these analyses did not further clarify the role of YdbH, YhjG, and YicH. In contrast, the three double mutants resulting from combining Δ*asmA*, Δ*tamB*, and Δ*yhdP* revealed key information. The Δ*asmA* allele behaved in an additive fashion with Δ*tamB* and Δ*yhdP* (Fig. 1C, Table S1A), indicating that AsmA performs a function that is different from those of TamB and YhdP. Previously, AsmA had been implicated in the folding of defective integral β-barrel OM proteins (OMPs) (37, 40, 41). Moreover, we uncovered negative synergistic effects between Δ*tamB* and Δ*yhdP*. Unlike the mildly defective single mutants, the Δ*yhdP* Δ*tamB* mutant showed severe OM permeability defects to bile salts and various antibiotics (Fig. 1C). Thus, our characterization suggests that while AsmA works independently, YhdP and TamB function in redundant fashion to maintain the barrier quality of the OM.

### TamA is required for TamB’s function.

TamB forms a complex with the OM β-barrel protein TamA in E. coli (Fig. 1A) (42). TamA is a homolog of BamA, the β-barrel component of the BAM complex that assembles β-barrel proteins in the OM (43, 44). In some organisms that lack TamA, TamB has been shown to interact with BamA instead (31, 38). The TAM complex has been proposed to function in the assembly of a subset of OM β-barrel proteins, including autotransporters and fimbrial ushers (29, 42, 45, 46). However, in some organisms, autotransporter biogenesis is not affected by the loss of TamB (47–49), and while biogenesis of autotransporters and ushers requires BAM, they are partially affected in *tamAB* mutants (46, 50). In addition, some bacteria lack autotransporters and fimbrial ushers but possess TamB (and BamA but not TamA) (31, 51). Therefore, the function of TamB remains unclear.

To determine if TamA is required for TamB’s function, we analyzed the effects of the Δ*tamA* allele and found that it causes the same phenotypes as Δ*tamB* by itself and in combination with Δ*yhdP* (Table S1B). Furthermore, plasmid-encoded TamB complements Δ*tamB* Δ*yhdP* but not Δ*tamA* Δ*yhdP* with respect to OM permeability defects (Fig. S2A). We therefore conclude that the OM protein TamA is required for TamB’s function in maintaining OM permeability.

10.1128/mBio.02714-21.2 FIG S2 TamA is required for TamB function. (A) Strains NR5161 (Δ*tamB* Δ*yhdP*) and NR3194 (Δ*tamA* Δ*yhdP*) are sensitive to bile salts and antibiotics and are indistinguishable under all conditions we tested. TamB encoded in plasmid pET23/42TamB restores resistance to bile salts and antibiotics when introduced into NR5161 but not NR3194. The pET23/42 control plasmid does not complement either strain. MacConkey (Mac) plates or LB plates containing 125 μg/ml ampicillin (resistance conferred by plasmids), 100 μg/ml bacitracin (Bac), 25 μg/ml erythromycin (Em), or 25 μg/ml vancomycin (Van) were photographed after overnight incubation at 37°C. (B) TamA is required for viability in the absence of YdbH and YhdP. Replacing the Δ*tamB*::*frt* allele of NR6834 (MG1655 Δ*tamB*::*frt* Δ*ydbH*::*frt tet2-3 yhdP*Ω-1::*bla araC* P_BAD_) with Δ*tamA*::*kan* resulted in strain NR7140 (MG1655 Δ*tamA*::*kan* Δ*ydbH*::*frt tet2-3 yhdP*Ω-1::*bla araC* P_BAD_), which is dependent on arabinose for growth like its parental strain NR6834. LB plates with and without arabinose (ARA) were streaked with NR6834 and NR7140 and incubated overnight at 37°C. Download FIG S2, PDF file, 0.6 MB. Copyright © 2021 Ruiz et al. 2021 Ruiz et al. https://creativecommons.org/licenses/by/4.0/ This content is distributed under the terms of the Creative Commons Attribution 4.0 International license.

### The combined loss of TamB and YhdP causes pleiotropic defects in the cell envelope.

**FIG 2:**
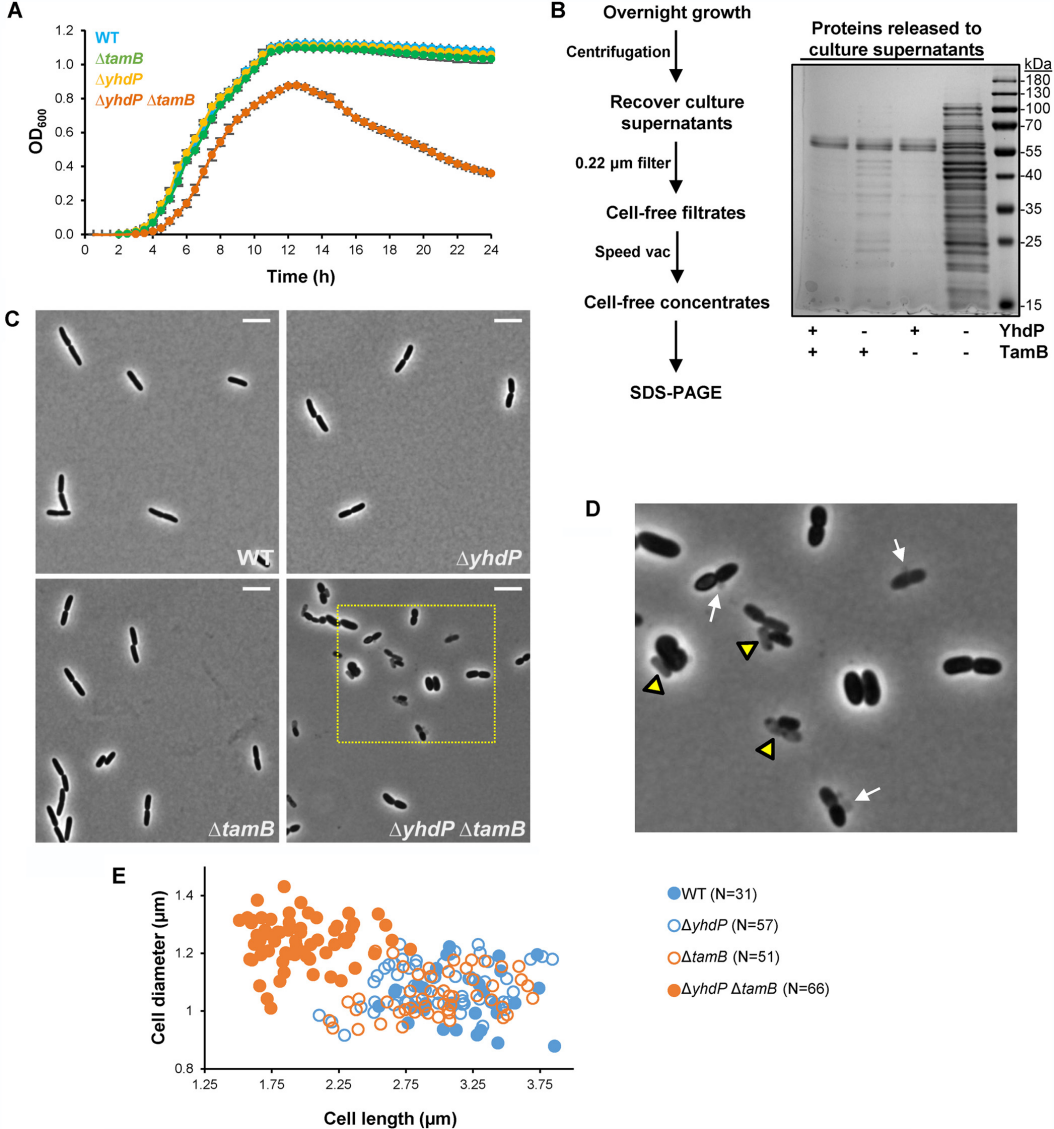
Combined loss of TamB and YhdP synergistically causes lysis and alterations to cellular morphology. (A) Cultures of the Δ*yhdP* Δ*tamB* mutant showed increased lysis, as demonstrated by a drop in optical density (OD_600_) when growing in LB at 37°C. Data represent the averages and standard deviations from three biological replicates. (B) On the left, scheme showing the experimental strategy to obtain cell-free culture supernatants from overnight cultures grown in LB at 37°C. On the right, SDS-polyacrylamide gel in which proteins were stained with Blue-BANDit after electrophoresis. Gel shown is representative of at least three independent experiments. (C) Phase-contrast microscopy of cells growing in LB at 37°C revealed that loss of either YhdP or TamB alone does not alter cell morphology, but loss of both factors leads to morphological defects. White scale bar (top right) represents 5 μm. Yellow rectangle marks area enlarged in panel D. (D) Δ*yhdP* Δ*tamB* cells exhibit pleiotropic morphological defects: decreased length and increased width, membrane vesiculation or blebbing (white arrows), and increased incidence of lysis (yellow triangles). Images are representative of at least three independent experiments. (E) Cells lacking both YhdP and TamB grow shorter and wider than the wild type and their respective single-mutant parents. The number of cells undergoing constriction after septation (with shape of the number 8) was observed with phase-contrast microscopy, and images were processed with ObjectJ (85) to measure cell length and width of each daughter cell undergoing constriction during exponential growth in LB at 37°C.

In addition to having OM permeability defects, we observed that cells lacking YhdP and TamB display other phenotypes indicative of severe defects in cell envelope biogenesis. While its parental single mutants grow similarly to the wild type at 37°C in LB, the Δ*yhdP* Δ*tamB* mutant exhibits increased lysis that becomes more evident upon entry into stationary phase (Fig. 2A). Indeed, analysis of concentrated cell-free filtrates from supernatants of overnight cultures showed higher protein content in samples from the Δ*tamB* Δ*yhdP* mutant than the wild-type strain (Fig. 2B). In contrast, samples from the Δ*yhdP* but not the Δ*tamB* single mutant only showed a slight increase in protein content. Furthermore, phase-contrast microscopy of exponentially growing cells showed that the increase in lysis in the Δ*tamB* Δ*yhdP* mutant is not limited to stationary-phase cultures. We could not detect lysis in wild-type and single-mutant cultures, but ghost (i.e., lysed) cells and membrane blebs were readily observed in Δ*tamB* Δ*yhdP* cultures (Fig. 2C and D). Phase-contrast microscopy also revealed that while the overall morphology (length and width) of wild-type and single-mutant cells is similar, Δ*yhdP* Δ*tamB* cells are more irregularly shaped, tending to be shorter and wider than the wild type and single-mutant parents (Fig. 2C to E).

We also observed that, on LB agar, the Δ*yhdP* Δ*tamB* mutant forms mucoid colonies, unlike the parent and wild-type strains (Fig. S3A). Mucoidy results when production of colanic acid surface capsule is upregulated by the Rcs envelope stress response via the RcsB response regulator in reaction to various signals, including cell surface stress (52). Capsule production involves the synthesis of intermediates that are linked to undecaprenyl-phosphate, an isoprenoid lipid carrier that is also required for the synthesis of the peptidoglycan cell wall (53). Since the cell wall determines cell shape and protects cells from osmotic lysis, we considered the possibility that lysis and altered cell shape in Δ*yhdP* Δ*tamB* cells could result from titration of undecaprenyl-phosphate from peptidoglycan synthesis by the upregulation of capsule production, as it has been reported when there is an imbalance in the production of polysaccharides whose synthesis rely on undecaprenyl-phosphate (54, 55). To test this possibility, we deleted *rcsB* in the Δ*yhdP* Δ*tamB* mutant. As expected, Δ*rcsB* eliminated mucoidy in the Δ*yhdP* Δ*tamB* mutant; however, the resulting triple mutant still shows increased lysis, severe OM permeability defects, and altered shape (Fig. S3). In fact, Δ*rcsB* worsens the growth of Δ*yhdP* Δ*tamB* cells. This detrimental effect appears to be solely caused by the effect that RcsB has on capsule production, since only deleting a gene responsible for capsule synthesis (*wcaJ*) conferred the same phenotype as Δ*rcsB* (Fig. S3). These results suggest that the Rcs system senses surface stress in Δ*yhdP* Δ*tamB* cells; in agreement, we found that their mucoidy requires the OM lipoprotein sensor RcsF since introduction of Δ*rcsF* abolishes their mucoid phenotype (52). Together, our results indicate that the loss of TamB and YhdP severely compromises the integrity of the cell envelope and that activation of the Rcs envelope-stress response confers some protection by inducing synthesis of a surface polysaccharide capsule.

10.1128/mBio.02714-21.3 FIG S3 Loss of colanic acid capsule impairs growth of the Δ*yhdP* Δ*tamB* mutant. (A) The Δ*tamB* Δ*yhdP* mutant, unlike its single mutant parents and the wild-type strain, is mucoid (photos of colonies bellow graph). In agreement, the Rcs envelope stress response is upregulated in the Δ*tamB* Δ*yhdP* double mutant. We constructed derivatives of a strain [MG1655 ΔlacZYA::FRT λimm 21 φ (P*rprA*'-*lacZ*^+^)] carrying a transcriptional *lacZ* fusion to the *rprA* promoter, which is upregulated by Rcs. Relative LacZ levels (normalized with respect to optical density) in various strains grown exponentially in LB and 37°C are shown. Data represent the average and standard deviation from three biological replicates. (B) The *wcaJ* gene is required for the synthesis of colanic acid capsule, and its expression is upregulated by RcsB, so the loss of either *wcaJ* or *rcsB* abolished mucoidy in the Δ*tamB* Δ*yhdP* double mutant. Introducing either a Δ*wcaJ* or a Δ*rcsB* null allele into the wild-type strain (WT) did not affect growth in LB at 37°C, as monitored by OD_600_. (C) Growth in LB and 37°C was determined by OD_600_ for strains MG1655 (wild type), NR5161 (Δ*tamB* Δ*yhdP*), NR6728 (Δ*tamB* Δ*yhdP* Δ*wcaJ*), and NR5200 (Δ*tamB* Δ*yhdP* Δ*rcsB).* The loss of either *wcaJ* or *rcsB* similarly compromises the growth of cells lacking TamB and YhdP. (D and E) Deletion of *rcsB* (D) or *wcaJ* (E) did not change the OM permeability of the wild-type or the Δ*tamB* Δ*yhdP* strains. Data represent the average and standard deviation from three biological replicates. If not shown, standard deviation equals zero. Download FIG S3, TIF file, 1.9 MB. Copyright © 2021 Ruiz et al. 2021 Ruiz et al. https://creativecommons.org/licenses/by/4.0/ This content is distributed under the terms of the Creative Commons Attribution 4.0 International license.

### Lysis of the Δ*tamB* Δ*yhdP* mutant results from defects in OM lipid homeostasis.

We have described that cells lacking TamB and YhdP exhibit phenotypes characteristic of OM biogenesis defects: increased permeability to bile salts and antibiotics, production of membrane blebs, and activation of the Rcs response. Not surprisingly, we found that the σ^E^ stress response is also activated in Δ*yhdP* Δ*tamB* cells (Fig. S4A). The σ^E^ system regulates synthesis of OM components and OM biogenesis factors in response to stresses, such as the misfolding of OMPs and off-pathway intermediates in LPS transport (56, 57). The OM contains two major types of proteins, OMPs, which fold into β-barrel structures, and lipoproteins, which are anchored to the OM via an N-terminal anchor (58, 59). OMP assembly is catalyzed by the β-barrel assembly machine (BAM), which is composed of the β-barrel protein BamA and the BamB-E lipoproteins (43, 60). Given that both BamA and BamD are essential for BAM function, OMP assembly itself also requires proper biogenesis of OMPs and OM lipoproteins. We therefore analyzed levels and folding of the major OMPs OmpA and OmpC to monitor BAM function and thereby the biogenesis of OMPs and OM lipoproteins. We could not detect misfolding of OmpA and OmpC in cells lacking YhdP and/or TamB (Fig. S4B), suggesting that these proteins do not function in either OMP or lipoprotein biogenesis. We did observe that the Δ*yhdP* Δ*tamB* mutant, unlike its mutant parents, produces higher levels of LPS than the wild type, which might be the cause for the activation of the σ^E^ stress response if some of the molecules go off pathway (Fig. S4C).

10.1128/mBio.02714-21.4 FIG S4 Loss of both TamB and YhdP activates the σ^E^ stress response and increases LPS levels. (A) The σ^E^ envelope stress response is induced in the *tamB* Δ*yhdP* double mutant, unlike in its ancestor derivatives of a strain [MG1655 ΔlacZYA::FRT λRS45 φ (P*rpoH3*'-*lacZ*^+^)] carrying a transcriptional *lacZ* fusion to the *rpoH3* promoter, which is upregulated by σ^E^. Relative LacZ levels (normalized with respect to the OD_600_) in various strains grown exponentially in LB at 37°C. Data represent the average and standard deviation from three biological replicates. (B) Whole-cell protein extracts obtained with BugBuster from exponentially growing cells (OD_600_, ∼0.6; LB, 37°C) were subjected to electrophoresis and immunoblotting for the periplasmic protease DegP (which is upregulated by the σ^E^ envelope stress response) and the β-barrel outer membrane proteins OmpA and OmpC. In unboiled samples, OmpA migrates in its folded conformation (∼25 kDa) and OmpC is not recognized by the antiserum. Boiling samples denatures and unfolds OmpA and OmpC, which migrate as indicated. An unidentified band above folded OmpA is marked with a question mark. The loss of YhdP and TamB increases the levels of DegP but has no detectable effect on the folding of OmpA and OmpC. (C) Samples were also subjected to electrophoresis and immunoblotting to compare levels of LPS. The loss of YhdP and TamB increases the levels of LPS. Intensity of the signal in the LPS band was measured, and values shown below the immunoblot represent relative values across samples that were calculated by setting levels in the wild-type strain MG1655 to 1.0. Immunoblots shown in panels B and C are representative of at least three independent experiments. (D) Effect of various additives to the growth of strains lacking TamB and/or YhdP. Growth curve of wild-type strain MG1655 (WT) and derivatives carrying Δ*tamB* and/or Δ*yhdP* alleles in LB at 37°C. When indicated above the graph, MgCl_2_ (to stabilize LPS on the cell surface), EDTA (to extract LPS from the cell surface), or the fatty acid oleic acid were added at the specified concentration at the beginning of the experiment. Data represent the average and standard deviation from three biological replicates. Download FIG S4, TIF file, 1.8 MB. Copyright © 2021 Ruiz et al. 2021 Ruiz et al. https://creativecommons.org/licenses/by/4.0/ This content is distributed under the terms of the Creative Commons Attribution 4.0 International license.

**FIG 3:**
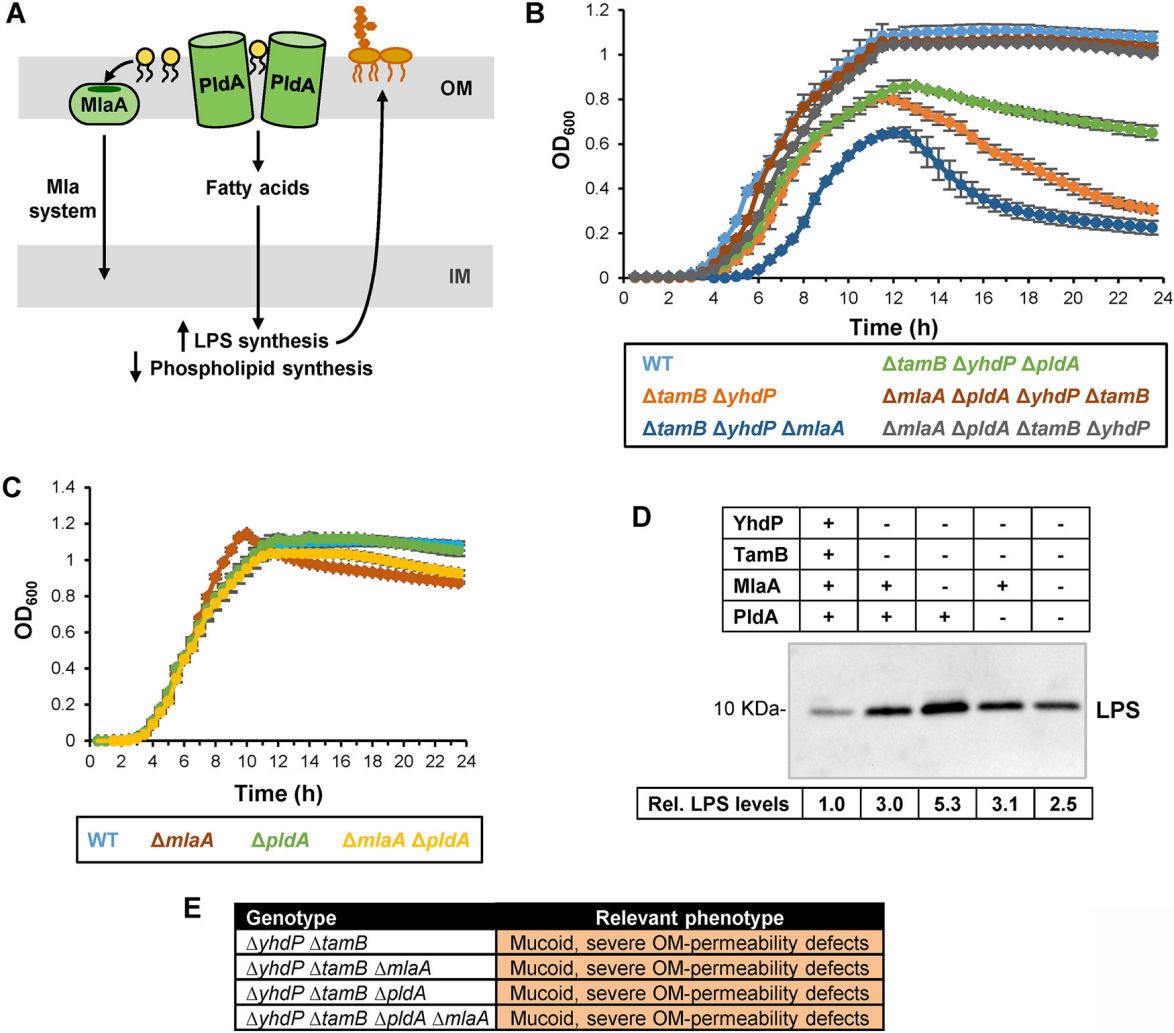
Lysis of the Δ*tamB* Δ*yhdP* mutant results from OM rupture and is suppressed by preventing the removal of phospholipids from the cell surface. (A) Cartoon depicting the function of MlaA and PldA. In wild-type cells, phospholipids mislocalized to the outer leaflet of the OM enter the Mla pathway through MlaA to be transported to the IM. Alternatively, mislocalized phospholipids can be hydrolyzed by a dimer of the PldA phospholipase. The released fatty acyl chains are recycled into the cytoplasm, where they induce higher production of LPS. (B) Growth curves of cultures growing in LB at 37°C. The lysis phenotype of the Δ*yhdP* Δ*tamB* double mutant is enhanced by Δ*mlaA* and partially suppressed by Δ*pldA*. Loss of both PldA and MlaA suppresses the lysis phenotype in the Δ*tamB* Δ*yhdP* mutant. Data represent averages and standard deviations from three biological replicates. (C) Growth curve of wild-type strain MG1655 (WT) and derivatives carrying Δ*mlaA* and/or Δ*pldA* alleles in LB at 37°C. Data represent the averages and standard deviations from three biological replicates. (D) Relative levels of LPS in the wild type and mutants lacking *tamB*, *yhdP*, *mlaA*, and/or *pldA* were measured using immunoblotting from whole-cell samples. Intensity of the signal in the LPS band was measured, and values shown below the immunoblot represent relative values across samples that were calculated by setting levels in the wild-type strain MG1655 to 1.0. (E) Table summarizing mucoidy and OM permeability defects in various strains. For detailed sensitivity data, refer to Table S1C. Data shown in panels D and E are representative of at least three independent experiments.

Elevated LPS levels and increased lysis during stationary phase resemble phenotypes previously reported for the mlaA* mutant (19). As stated earlier, the dominant-negative MlaA* variant causes the mislocalization of phospholipids to the cell surface, which activates PldA, leading to an increase in LPS synthesis and a decrease in phospholipid synthesis (Fig. 3A) (23). The resulting PldA-dependent imbalance in OM lipid synthesis leads to loss of OM material through vesiculation and eventually results in lysis in stationary phase because cells cannot synthesize enough lipids to overcome the loss of OM material (19, 23). Here, we sought to investigate if the stationary-phase-induced lysis in Δ*yhdP* Δ*tamB* mutants was related to defects in the OM. Loss of OM material in mlaA* cells can be partially suppressed by the addition of Mg^2+^ (19). Similarly, adding 1 mM MgCl_2_ to the growth medium partially reduces lysis in Δ*yhdP* Δ*tamB* cultures (Fig. S4D), suggesting that lysis in the Δ*yhdP* Δ*tamB* mutant results from the loss of OM material. If so, we expected that Δ*yhdP* Δ*tamB* cells would be hypersensitive to EDTA, since this chelator removes LPS from the cell surface and causes phospholipids to translocate to the outer leaflet of the OM (61). Indeed, the Δ*yhdP* Δ*tamB* mutant is hypersensitive to EDTA (MIC of 0.195 mM compared to 25 mM in the wild-type and single-mutant parents). We also found that adding subinhibitory amounts of EDTA (0.5 mM) to cultures of wild-type and Δ*yhdP* and Δ*tamB* single-mutant strains triggers lysis in stationary phase (Fig. S4D), which is more pronounced in the single mutants than in the wild type. We next explored if MlaA and PldA play a role in the lysis phenotype of Δ*yhdP* Δ*tamB* cells. While deleting *mlaA* increased lysis, deleting *pldA* decreased the rate and extent of lysis induced during stationary phase in Δ*yhdP* Δ*tamB* cells (Fig. 3B and C).

It seemed paradoxical that removing MlaA would have the opposite effect of removing PldA, given that the individual loss of each factor increases the amount of phospholipids at the cell surface. We reasoned that the increase in lysis of Δ*yhdP* Δ*tamB* cells caused by the loss of MlaA might be dependent on PldA. Specifically, Δ*mlaA* should increase the amount of PldA’s substrate (i.e., mislocalized phospholipids), which would upregulate LPS levels while decreasing phospholipid synthesis (23). Indeed, Δ*mlaA* further increases the already elevated LPS levels in a Δ*yhdP* Δ*tamB* mutant (Fig. 3D). We therefore built a Δ*mlaA* Δ*pldA* Δ*yhdP* Δ*tamB* mutant. Strikingly, the combined loss of Δ*mlaA* and Δ*pldA* fully suppresses lysis of Δ*yhdP* Δ*tamB* cells, yielding a wild-type growth pattern (Fig. 3B). As expected, the Δ*mlaA* Δ*pldA* Δ*yhdP* Δ*tamB* mutant also has lower LPS levels than the Δ*yhdP* Δ*tamB* Δ*mlaA* mutant. Nevertheless, the quadruple mutant remains sensitive to bile salts and antibiotics (Fig. 3E, Table S1C).

Together, our results suggest that Δ*yhdP* Δ*tamB* cells have OM structural defects that somehow lead to increased levels of phospholipids in their cell surface, which results in increased sensitivity to bile salts and antibiotics and activation of the Mla and PldA pathways. In Δ*yhdP* Δ*tamB* cells, both MlaA and PldA contribute to lysis by removing these mislocalized phospholipids. PldA’s action is particularly detrimental to Δ*yhdP* Δ*tamB* cells because it induces LPS synthesis, causing a further imbalance in OM lipid composition (23). In agreement, adding fatty acids (oleic acid) to the medium exacerbates the lysis phenotype in Δ*yhdP* Δ*tamB* cultures, although a detergent-like effect could also contribute to or cause lysis in these mutant cells (Fig. S4D). If only MlaA is removed, Δ*yhdP* Δ*tamB* cells lyse even more because of PldA’s upregulation of LPS levels; however, when both PldA and MlaA are removed, lysis is abolished and cells can grow like the wild type. Thus, these results suggest that TamB and YhdP are required for OM lipid homeostasis.

### The AsmA-like protein family is essential for viability in E. coli.

The main lipid constituents of the OM are LPS and phospholipids. Notably, the Gram-negative *Borrelia* does not produce LPS, yet it encodes *tamB*, which appears to be essential (38). In addition, one of us (N. Ruiz) previously took advantage of the reduced size of two Gram-negative endosymbionts carrying <600 genes to search for envelope biogenesis factors and found *tamB* present in both despite one of these bacteria not having LPS biogenesis genes (51). Here, we expanded this search to five additional gammaproteobacterial endosymbionts and to YhdP. We still found no correlation between the presence of TamB and YhdP and that of LPS biogenesis proteins (Table S2), ruling out a direct role for TamB and YhdP in LPS biogenesis. The fact that these endosymbionts encode either *tamB* or *yhdP* even after having undergone massive gene loss strongly suggests that these proteins perform a crucial function in the physiology of these bacteria. Given that YhdP has been implicated in IM-to-OM transport of phospholipids (18), and its predicted protein architecture supports this proposed role (Fig. S1) (27, 30), the simplest explanation of the evidence presented so far here and by others is that YhdP and TamB mediate phospholipid transport between the IM and OM. However, anterograde phospholipid transport is expected to be essential for building the OM, but even the combined loss of YhdP and TamB is not lethal. We therefore explored if the functional redundancy between TamB and YhdP extends to additional AsmA-like paralogs.

10.1128/mBio.02714-21.7 TABLE S2 Conservation of TamB and YhdP homologs in endosymbionts does not correlate with LPS production. Download Table S2, PDF file, 0.1 MB. Copyright © 2021 Ruiz et al. 2021 Ruiz et al. https://creativecommons.org/licenses/by/4.0/ This content is distributed under the terms of the Creative Commons Attribution 4.0 International license.

**FIG 4:**
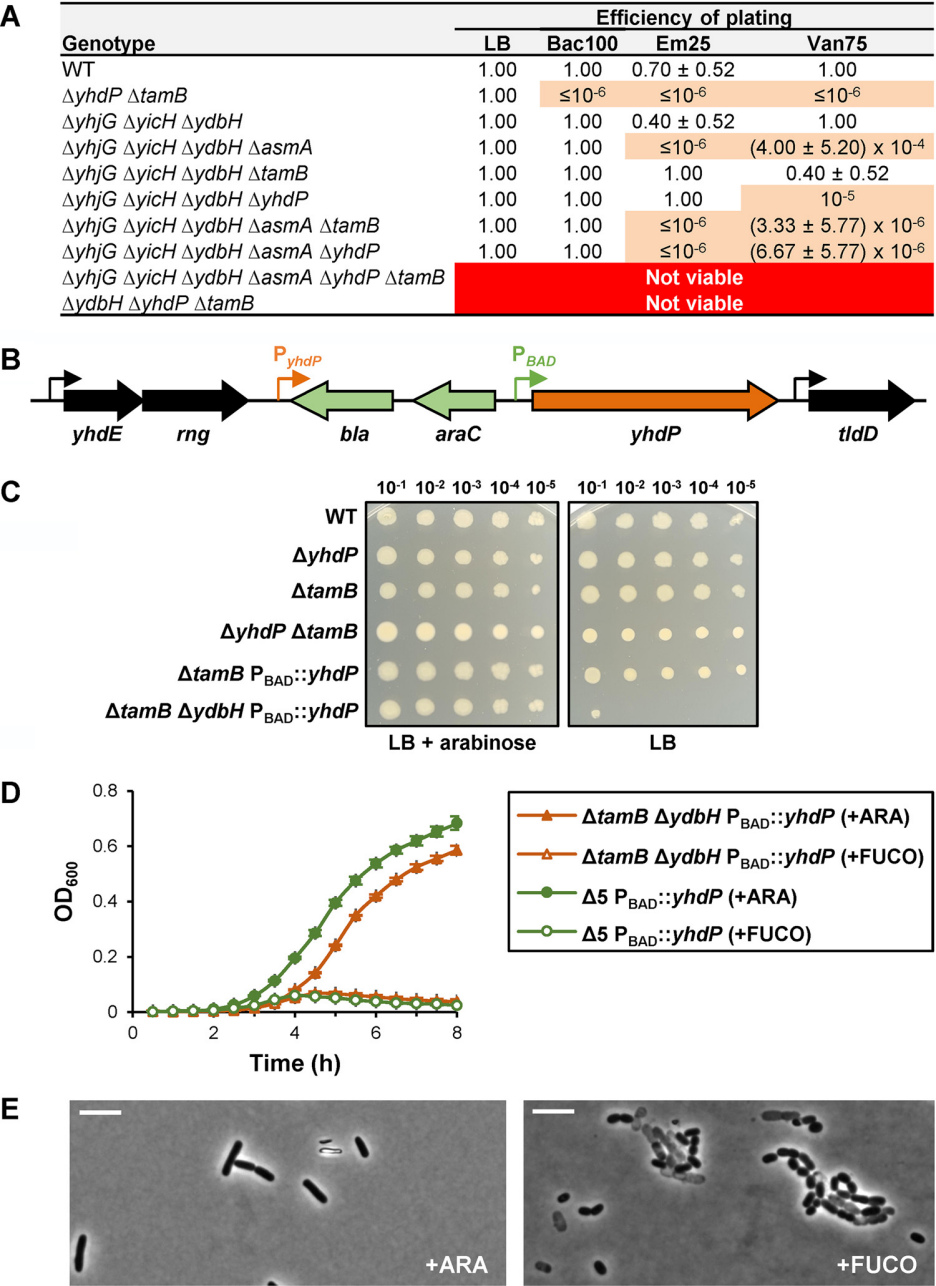
TamB, YhdP, and YdbH are redundant proteins that perform an essential function in E. coli. (A) Table showing synthetic genetic interactions resulting from combining deletion alleles of AsmA-like factors. The permeability defects of quintuple mutants only producing either TamB or YhdP resemble those of combining the loss of AsmA and either TamB or YhdP (Fig. 1 and Table S1A). The loss of all AsmA-like factors or the combined loss of TamB, YhdP, and YdbH is lethal. Data represent the average and standard deviation from three biological replicates. If not shown, standard deviation equals zero. (B) Chromosomal *yhdP* locus in YhdP depletion strains. Sequence encoding *bla-araC-P_BAD_* was inserted upstream of *yhdP* to decouple it from its native promoter (P_yhdP_). The resulting recombinant locus has been engineered to have *yhdP* transcription under the arabinose-dependent activator AraC. (C) Cultures grown in LB with arabinose overnight at 37°C were diluted 1:10 from left to right and then stamped with a pin replicator onto LB agar containing or lacking arabinose. Growth of the Δ*ydbH* Δ*tamB* P_BAD_::*yhdP* mutant is arabinose dependent. (D) Depletion of YhdP in a quintuple (Δ5) or a Δ*ydbH* Δ*tamB* double mutant is lethal. Overnight cultures of NR5921 (MG1655 Δ*tamB*::*frt* Δ*ydbH*::*kan yhdP*Ω-1::*bla araC* P_BAD_) and NR5850 (MG1655 Δ*yhjG*::*frt* Δ*yicH*::*frt* Δ*ydbH*::*frt* Δ*asmA*::*frt* Δ*tamB*::*kan yhdP*Ω-1::*bla araC* P_BAD_) were grown in LB with arabinose at 37°C. After a 1:5,000 dilution in LB with arabinose (+YhdP) or fucose (YhdP depletion), growth at 37°C was measured by monitoring the OD_600_. Data represent the average and standard deviation from three biological replicates. (E) Phase-contrast microscopy (100× objective) of strain NR5921 grown in the presence of arabinose or fucose. White scale bar represents 5 μm. Data are representative of at least three independent experiments.

We constructed mutants lacking more than two AsmA-like proteins. Although our data (Fig. 1) suggest that AsmA functions independently of YhdP and TamB, we still included AsmA in our studies. We first combined Δ*yhjG*, Δ*yicH*, and Δ*ydbH* because they do not confer defects individually or in pairs and then introduced Δ*asmA*, Δ*yhdP*, and Δ*tamB*, which on their own confer OM permeability defects. After constructing several mutants, we assayed their OM permeability and compared them to single and double mutants (Fig. 4A, Table S1A) and found that we could not generate a mutant lacking all six AsmA-like proteins (Fig. 4A), suggesting that the AsmA-like family of proteins are essential for viability in E. coli.

### TamB, YhdP, and YdbH are redundant proteins essential for viability in E. coli.

Since our genetic analyses indicated that TamB/YhdP and AsmA function in different pathways, we hypothesized that the synthetic lethality observed when attempting to construct a strain lacking all AsmA-like proteins could result from redundancy between TamB/YhdP and YdbH, YicH, and/or YhjG. We found that while the Δ*tamB* Δ*yhdP* Δ*yicH* and Δ*tamB* Δ*yhdP* Δ*yhjG* triple mutants were viable and phenotypically indistinguishable from the Δ*tamB* Δ*yhdP* mutant, we could not build the Δ*tamB* Δ*yhdP* Δ*ydbH* triple mutant (Fig. 4A), suggesting these three alleles are synthetic lethal.

We next constructed a YhdP depletion strain to better test if Δ*tamB*, Δ*yhdP*, and Δ*ydbH* are synthetically lethal. By altering its promoter region, we placed *yhdP* transcription under the control of the arabinose-inducible promoter P_BAD_ (Fig. 4B) (62). We confirmed that expression of *yhdP* is controlled by arabinose in strains carrying P_BAD_::*yhdP* by showing that a Δ*tamB* P_BAD_::*yhdP* strain exhibits OM permeability defects similar to those of a Δ*tamB* Δ*yhdP* mutant when grown in the presence of the anti-inducer d-fucose (an l-arabinose analog) but not in the presence of the inducer arabinose (Fig. S5A and B). Next, we constructed a Δ*tamB* Δ*ydbH* P_BAD_::*yhdP* strain in the presence of arabinose and determined that its growth is arabinose dependent (Fig. 4C and D). Thus, Δ*tamB*, Δ*yhdP*, and Δ*ydbH* are indeed synthetically lethal. We also built a YhdP depletion strain lacking the genes encoding the other five AsmA-like proteins and determined that it behaves similarly to the Δ*tamB* Δ*ydbH* P_BAD_::*yhdP* strain (Fig. 4D). Further characterization of the Δ*tamB* Δ*ydbH* P_BAD_::*yhdP* strain showed that growth in the presence of d-fucose does not cause defects in the assembly of the major β-barrel protein OmpA but induces production of DegP, which is controlled by the σ^E^ stress response (56), and LPS (Fig. S5). Phase-contrast microscopy revealed that, in the presence of arabinose, the Δ*tamB* Δ*ydbH* P_BAD_::*yhdP* strain cells appear like wild-type rods, but, in the presence of fucose, the depletion strain undergoes lysis and exhibits morphological defects (Fig. 4E).

10.1128/mBio.02714-21.5 FIG S5 Phenotypic analysis of the Δ*tamB* Δ*ydbH* YhdP depletion strain. (A) Relevant genetic features of strain NR5921 (MG1655 Δ*tamB*::*frt* Δ*ydbH*::*kan yhdP*Ω-1::*bla araC* P_BAD_). (B) MIC assay revealed that depletion (+Fuco) of YhdP in a Δ*tamB* mutant increases sensitivity to bacitracin and vancomycin with respect to YhdP-replete conditions (+Ara), similar to that observed in a Δ*tamB* Δ*yhdP* mutant. (C) Growth of YhdP depletion strain NR5921 at 37°C in LB in the presence of arabinose (ARA) or fucose (FUCO) to induce or repress, respectively, expression of *yhdP*. An overnight culture of NR5921 was grown in LB in the presence of arabinose at 37°C. After a 1:5,000 dilution in LB containing either arabinose or fucose, growth at 37°C was measured by monitoring the OD_600_. Depletion of YhdP in the Δ*tamB* Δ*ydbH* mutant arrests growth. Cells were collected at points labelled A1, F1, and F2 to prepare whole-cell extracts for immunoblotting shown in panels D and E. (D) Whole-cell protein extracts obtained with BugBuster (at times indicated in panel C) were subjected to electrophoresis and immunoblotting for the periplasmic protease DegP (which is upregulated by the σ^E^ envelope stress response) and the β-barrel outer membrane protein OmpA. In unboiled samples, OmpA migrates in its folded conformation (∼25 kDa), but boiling samples denatures and unfolds OmpA, which migrates more slowly as indicated. An unidentified band above folded OmpA is marked with a question mark. Depletion of YhdP in the Δ*tamB* Δ*ydbH* mutant increases the levels of DegP but has no detectable effect on the folding of OmpA. (E) Samples in panel D were also subjected to electrophoresis and immunoblotting to compare levels of LPS. Depletion of YhdP increases the levels of LPS and leads to the appearance of a band of slightly higher mass (marked with asterisk) that likely results from LPS being modified with colanic acid because of upregulation of colanic acid production and/or accumulation of LPS at the IM (supplemental material text references 14 and 15). Data shown are representative of at least three independent experiments. (F) The combined loss of PldA and MlaA does not suppress the essentiality of TamB, YhdP, and YdbH. Introducing the Δ*pldA* Δ*mlaA* alleles into the YhdP-depletion strain lacking TamB and YdbH, NR6834 (MG1655 Δ*tamB*::*frt* Δ*ydbH*::*frt tet2-3 yhdP*Ω-1::*bla araC* P_BAD_), does not suppress dependence on the inducer arabinose for growth. LB plates with and without arabinose (ARA) were streaked with NR6834 and NR7030 (MG1655 Δ*tamB*::*frt* Δ*ydbH*::*frt* Δ*pldA*::*frt* Δ*mlaA*::*kan tet2-3 yhdP*Ω-1::*bla araC* P_BAD_) and incubated overnight at 37°C. Download FIG S5, TIF file, 2.9 MB. Copyright © 2021 Ruiz et al. 2021 Ruiz et al. https://creativecommons.org/licenses/by/4.0/ This content is distributed under the terms of the Creative Commons Attribution 4.0 International license.

Given that the combined loss of *mlaA* and *pldA* suppresses lysis in Δ*tamB* Δ*yhdP* cells, we tested if it could also suppress the dependence on arabinose for growth of the Δ*tamB* Δ*ydbH* P_BAD_::*yhdP* strain. We found that it could not (Fig. S5F). However, we showed that TamA is essential in cells lacking YhdP and YdbH, confirming our earlier conclusion that TamA is required for TamB function (Fig. S2B). Altogether, our data demonstrate that TamB, YhdP, and YdbH are redundant in performing a function that is essential for growth of E. coli. Nevertheless, the difference in phenotypes observed in the three Δ*tamB*, Δ*yhdP*, and Δ*ydbH* single mutants, and their corresponding double and triple mutants, also suggest that despite being functionally redundant, these proteins are not equivalent (Table S1A).

### Gain-of-function substitutions in YdbH suppress defects in the Δ*tamB* Δ*yhdP* mutant.

We next searched for suppressor mutations that could provide information about the function of TamB, YhdP, and YdbH using both reverse and forward genetics. We focused on isolating suppressors of the Δ*tamB* Δ*yhdP* double mutant. For the reverse-genetic approach, we tested whether the loss of the enterobacterial common antigen (ECA) caused by deleting *wecA* could suppress the increase in OM permeability and/or lysis, since, as stated earlier, it suppresses OM permeability defects in a Δ*yhdP* mutant (25). We did not observe changes in OM permeability in either the wild-type or the Δ*tamB* mutant, but, as previously reported, observed that Δ*wecA* suppresses the sensitivity of a Δ*yhdP* mutant to vancomycin (Table S1D) (25). However, Δ*wecA* does not suppress the OM permeability defects in the Δ*tamB* Δ*yhdP* mutant and, in fact, severely compromises its growth (Table S1D and data not shown). Thus, the ability of the loss of ECA to suppress the OM permeability defects caused by Δ*yhdP* requires TamB, and losing ECA has a detrimental effect on the fitness of Δ*tamB* Δ*yhdP* cells.

**FIG 5:**
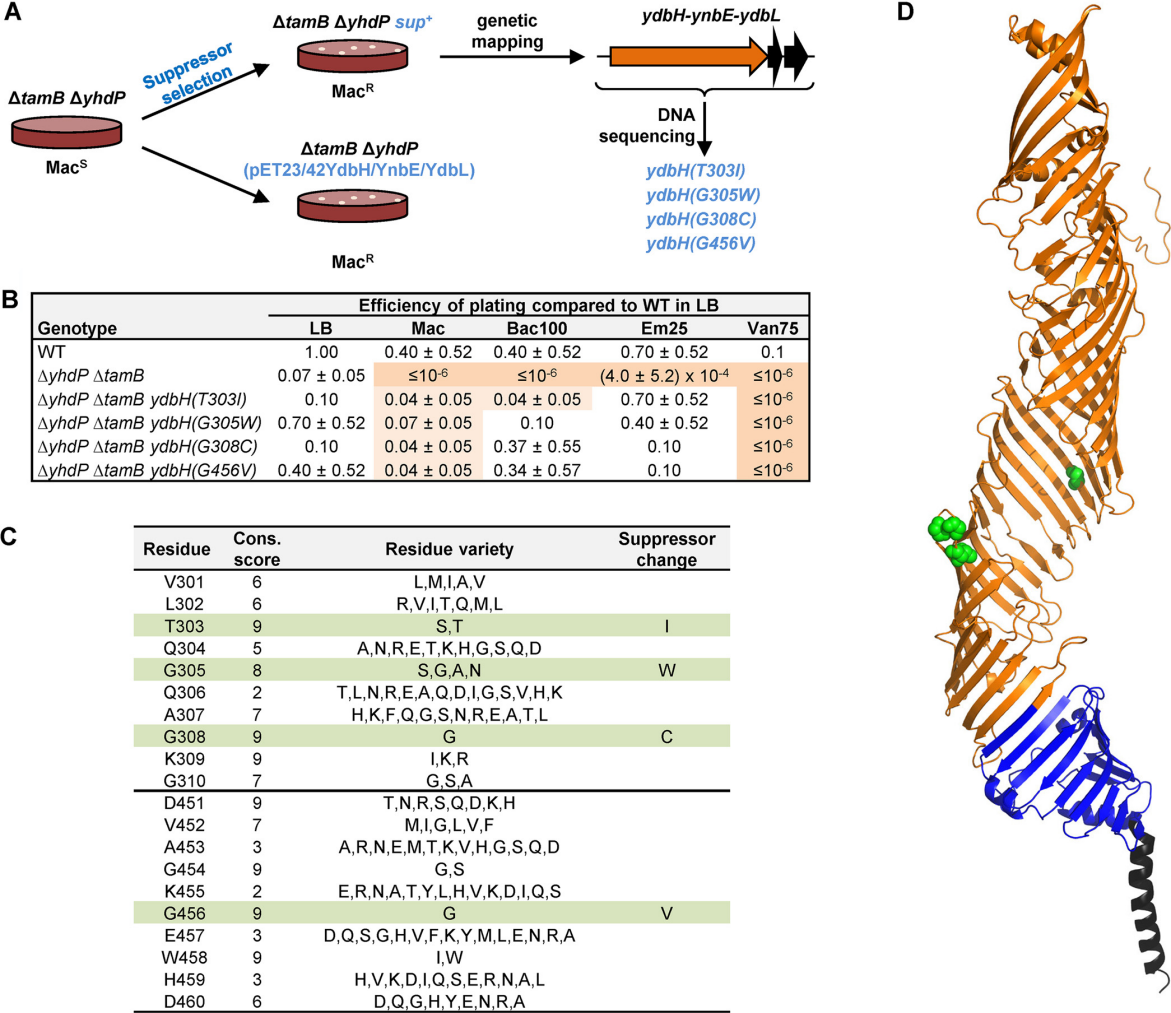
Gain-of-function substitutions in YdbH suppress defects in the Δ*tamB* Δ*yhdP* mutant. (A) Suppressors of the sensitivity of the Δ*tamB* Δ*yhdP* double mutant to bile salts were selected on MacConkey (Mac) agar. Suppressor mutations are missense mutations in *ydbH*. Introduction of a plasmid carrying the *ydbH-ynbE-ydbL* locus into the Δ*tamB* Δ*yhdP* mutant also suppresses its inability to grow on MacConkey agar. (B) Missense mutations in *ydbH* suppress OM permeability defects in the Δ*tamB* Δ*yhdP* double mutant. Data represent the average and standard deviation from three biological replicates. If not shown, standard deviation equals zero. (C) Suppressor mutations in *ydbH* alter residues that are highly conserved among 288 unique YdbH homologs. Table shows Consurf (86) conservation scores (Cons. score) of residues in the regions where suppressing changes are localized in YdbH. Conservation scale ranges from 1 (most variable) to 9 (most conserved). Although these residues are conserved among YdbH orthologs, they are not conserved at the same positions in YhdP and TamB. (D) Model structure (see Fig. S1) of YdbH showing residues altered by suppressor mutations as green spheres. The cluster containing residues 303, 305, and 308 is located in a loop, while the side chain of residue 456 faces the interior of the tubular AsmA-like domain.

We next employed an unbiased selection for spontaneous mutants that restore growth of the Δ*tamB* Δ*yhdP* mutant in the presence of bile salts (i.e., MacConkey agar). After mapping suppressor mutations by genetic linkage, we identified four different missense mutations in *ydbH* that change conserved residues in the periplasmic region of YdbH, T303I, G305W, G308C, and G456V (Fig. 5). We found that these missense *ydbH* alleles also decrease sensitivity to hydrophobic antibiotics, but not vancomycin (Fig. 5B). Thus, these suppressor mutations do not fully restore wild-type phenotypes. Given that the loss of YdbH is lethal in the Δ*tamB* Δ*yhdP* mutant, we expected these alleles to be gain-of-function alleles. In agreement with these alleles being gain of function and not loss of function, introducing a plasmid carrying the wild-type *ydbH* locus also restores growth of the Δ*tamB* Δ*yhdP* mutant on MacConkey agar. Thus, altering YdbH function through mutations or increasing levels of wild-type YdbH function suppresses sensitivity to bile salts (Fig. 5A). Further characterization will be needed to understand how these changes suppress defects in Δ*tamB* Δ*yhdP* cells, but they support our conclusion that TamB, YhdP, and YdbH are functionally redundant but not equivalent.

## DISCUSSION

The AsmA-like family of proteins has been recognized as conserved and specific to didermic Gram-negative bacteria, but determining its function has been difficult. The fact that many organisms, including E. coli, the model bacterium for studying the Gram-negative envelope, encode up to six AsmA-like paralogs (TamB, YhdP, YdbH, AsmA, YicH, and YhjG) has limited progress for our understanding of this protein family (31). Indeed, although previous studies have linked AsmA, TamB, and YhdP to envelope biogenesis in various bacteria, their role remains mostly uncharacterized (18, 24, 25, 28, 37–42, 45, 47–49). To investigate whether redundancy was occluding their function in E. coli, we used reverse genetics to generate and characterize mutants lacking one or several AsmA-like proteins. Our work demonstrates that TamB, YhdP, and YdbH are redundant in performing a function that is critical for OM lipid homeostasis and essential for growth.

**FIG 6:**
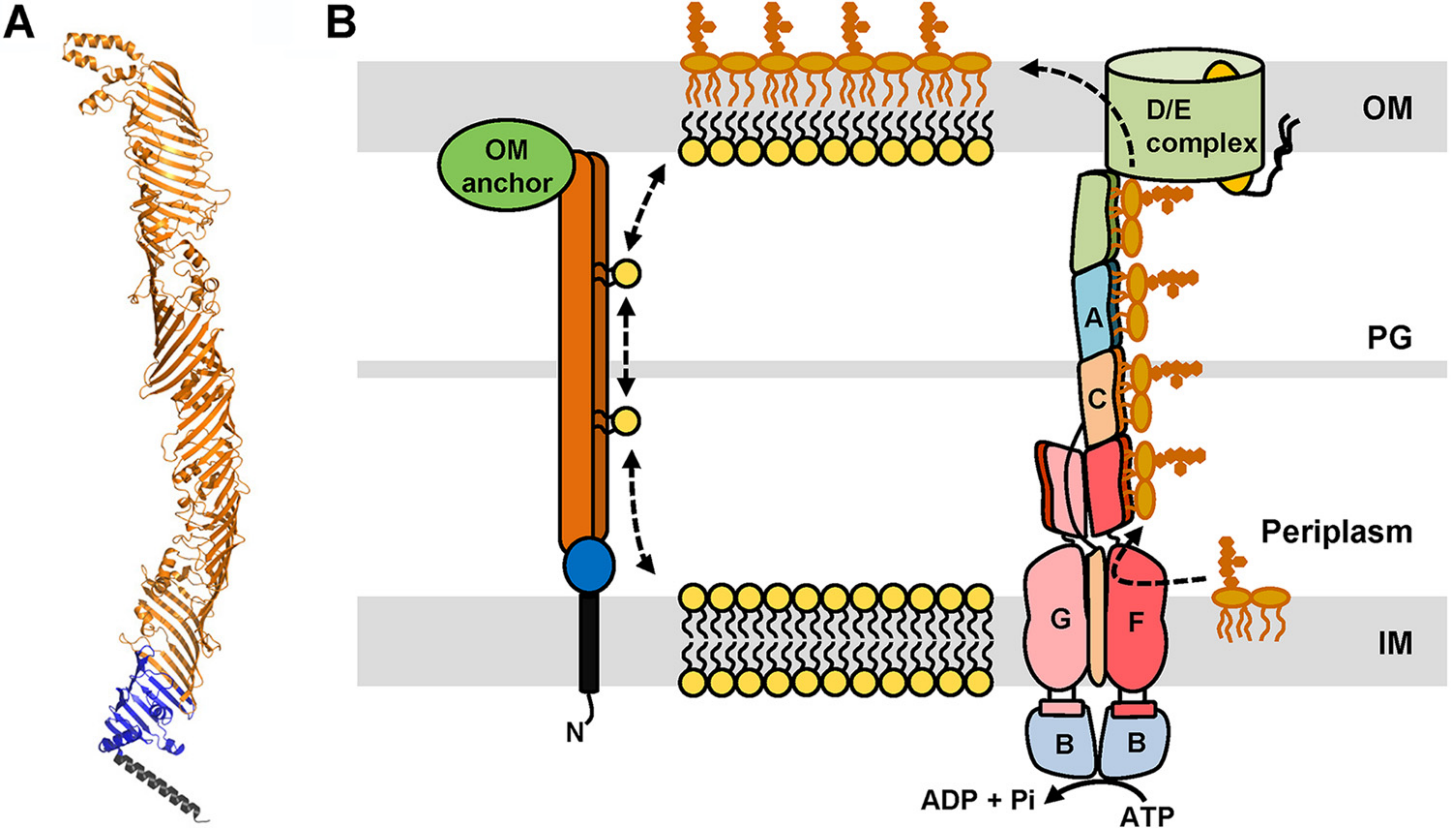
Model of OM lipid assembly. (A) Cartoon representation of the structural prediction for YhdP by AlphaFold (32). Regions of the structural model are colored as in Fig. 1A, with the transmembrane helix in black, the chorein-N domain in blue, and the AsmA domain in orange. (B) Model of OM lipid transport and assembly. The Lpt system (on the right) transports newly synthesized LPS molecules from the IM to the outer leaflet of the OM. We propose that TamB, YhdP, and YdbH transport phospholipids between the IM and OM. In this model, TamB, YhdP, and YdbH physically bridge the IM and OM by anchoring to the IM through their N-terminal α-helix (black segment) and likely to the OM via interactions with partners (green oval). The predicted structure of the large periplasmic region of these proteins would form a structure similar to the Lpt bridge and provide protection to the hydrophobic fatty acid chains of phospholipids as they travel through the periplasm. LPS transport is unidirectional and powered by ATP, while bidirectional phospholipid transport would be driven by diffusion through TamB, YhdP, and YdbH.

Previously, YhdP had been implicated in diffusive anterograde phospholipid transport between the IM and OM in mlaA* E. coli mutants, which have altered OM lipid structure (18). However, its role in anterograde phospholipid transport in wild-type cells remained unclear, especially because the loss of YhdP results in mild phenotypes but anterograde phospholipid transport is presumed to be essential for building the OM and thereby growth (24, 25). Our work addresses these issues by showing that the combined loss of YhdP, TamB, and YdbH is lethal in an otherwise wild-type strain because these proteins are crucial in maintaining lipid homeostasis at the OM (Fig. 3 and 4). This essential role for growth is also underscored by the fact that at least one of these proteins is conserved in Gram-negative endosymbionts that encode fewer than 600 proteins in their reduced-size genomes (Table S2) (51). Importantly, TamB, YhdP, and YdbH are homologous to the eukaryotic proteins Vps13 and Atg2, which were recently shown to constitute a new family of lipid transporters at membrane-contact sites between organelles (33–35, 63), and the less-characterized TIC236 and Mdm31/32 homologs, which are also needed for proper biogenesis of chloroplasts and mitochondria, respectively (27, 30, 33–35, 63–65). Given this body of evidence and that anterograde IM-to-OM phospholipid transport is the only essential process required for OM biogenesis that is yet to be linked to the essentiality of any protein(s) (1), we propose that TamB, YhdP, and YdbH transport phospholipids between the IM and OM (Fig. 6).

Transport of phospholipids between the IM and OM has been proposed to occur by diffusion mediated either by proteinaceous bridge-like structures or by lipid bilayer structures fusing the IM and OM (1, 9, 13, 18, 19, 66, 67). In the latter model, TamB, YhdP, and YdbH could be responsible for building or maintaining the proposed membrane bridges or for preventing the flow and mislocalization of proteins or lipid-linked molecules between the IM and OM. However, based on the predicted architecture of their homologs, Vps13 and Atg2, and the structural models recently released by AlphaFold (Fig. S1) (32), we favor a model in which TamB, YhdP, and YdbH are proteinaceous bridges between the IM and OM that directly facilitate the flow of phospholipids (Fig. 6). Vps13 and Atg2 are large intermembrane bridge proteins that are anchored to the membrane of one organelle via an N-terminal α-helix; this anchor is then followed by a chorein-N domain, a large region rich in β-strands, and variable domains that are thought to mediate interactions with different factors at the membrane of another organelle (30, 33, 63, 64, 68). The large region rich in β-strands bridging the two membranes is modeled to fold into a structure that resembles the periplasmic bridge formed by the Lpt proteins that transports the glycolipid LPS from the IM to the OM (11, 69, 70) (Fig. 6). Specifically, they are thought to form an elongated tube-like structure with a lateral opening along the long axis that leads to a hydrophobic groove that interacts with phospholipids by protecting the hydrophobic fatty acid tails of multiple lipids as they travel through aqueous compartments from one membrane to another (33, 68). TamB, YhdP, and YdbH also have an N-terminal α-helix that is followed by a chorein-N domain and a large portion rich in β-strands (>700 amino acids [aa]) large enough to cross the periplasm (27). Their structure is predicted to resemble that of Atg2 (Fig. S1). The eukaryotic homologs Vps13 and Atg2 contact two membranes from different organelles and associate with various partners through their C-terminal domains (30, 64). To date, no partners have been identified for YhdP and YdbH, but TamB interacts, depending on the organism, with the OMP TamA or BamA (31, 38, 42). In agreement, our data show that TamA is required for TamB’s function (Table S1B and Fig. S2). Based on the TamB-TamA interaction and that of their eukaryotic homologs with their partners, we propose that YhdP and YdbH are also likely to require an OM anchor (Fig. 6). We therefore suggest that TamB, YhdP, and YdbH constitute the membrane-contact sites proposed decades ago to mediate phospholipid transport between the IM and OM (66, 67, 71). In this model, TamB, YhdP, and YdbH would provide a bridge-like structure resembling that of the Lpt bridge that protects phospholipids as they travel across the periplasm (Fig. 6). However, unlike the unidirectional LPS transport mediated by the Lpt system, TamB, YhdP, and YdbH would support the previously reported bidirectional diffusive flow of phospholipids (Fig. 6) (9, 18, 19, 66, 67).

Functional redundancy between TamB, YhdP, and YdbH would also explain why the identification of the mechanism for anterograde phospholipid transport has remained elusive despite the great progress made in the identification and characterization of factors involved in the biogenesis of other envelope components (1, 13). Nevertheless, even though our data show that TamB, YhdP, and YdbH are redundant and any of them is sufficient to support growth of E. coli, these proteins are not functionally equivalent. Instead, our phenotypic analysis of the respective single and double mutants has revealed a functional hierarchy in the cell envelope that correlates with protein size (Fig. 1): TamB and YhdP play a more similar and important role than YdbH. The nature of this specialization is unknown, but possible explanations include differences in expression, cargo preference, and/or cellular localization as it occurs among the four human Vps13 paralogs (64). It is also possible that the specialization of these paralogs results from additional different functions that these proteins have evolved to perform. For example, TamB has been shown to affect the transport of a nonconserved subset of β-barrel proteins from the IM to the OM (28, 29, 42, 46). Whether this is an additional function of TamB or a secondary effect of the loss of its primary role in OM lipid biogenesis needs to be determined.

Lastly, we note that growth of the OM lipid bilayer requires the balanced assembly of phospholipids at the inner leaflet and of LPS and other lipid-linked oligosaccharides at the outer leaflet of the OM (1). How the synthesis, transport, and assembly of these lipid components are coordinated is poorly understood, but studies like ours and those previously done on mlaA* show that imbalance leads to severe defects in asymmetry and even death when the growth of the two leaflets of the OM is out of balance (18, 19). In mlaA* mutants, growth of the inner leaflet of the OM is compromised because of the aberrant translocation of phospholipids to the cell surface, which, in turn, causes the activation of PldA and downstream upregulation of LPS synthesis. These two events increase the synthesis of the main lipid component of the outer leaflet (LPS) and decrease the presence and synthesis, respectively, of the main lipid component of the inner leaflet (phospholipids). In our study, we found that a Δ*tamB* Δ*yhdP* mutant undergoes lysis that is suppressed by the loss of MlaA and PldA (Fig. 3). We propose that the compromised flow of phospholipids to the OM in the Δ*tamB* Δ*yhdP* mutant disrupts OM lipid homeostasis, somehow also leading to the activation of PldA and the subsequent upregulation of LPS synthesis. Upregulating the production of the main component of the outer leaflet of the OM (LPS) likely harms the cell through a futile cycle in which the LPS-driven growth of the outer leaflet of the OM would increase the demand for an already compromised transport of phospholipids, further driving the disruption of OM lipid homeostasis. How phospholipids are translocated across the OM in wild-type and Δ*tamB* Δ*yhdP* cells also remains unknown. Recent studies are just beginning to reveal how OM lipid asymmetry and LPS transport may be sensed by PldA and YejM to coordinate the synthesis of phospholipids and LPS through the regulated degradation of the LPS synthesis enzyme LpxC by FtsH/LepB (72–77). Our work calls for investigating how TamB, YhdP, and YdbH may be integrated in these systems, as well as the role that other surface components such as ECA and capsule may play.

## MATERIALS AND METHODS

### Bacterial strains.

Strains were derived from wild-type strain MG1655 (78) and are listed in Table S3A in the supplemental material. Deletion alleles were derived from the Keio collection (79) and introduced into the appropriate strains by generalized P1vir transduction and selection for kanamycin resistance (80). When necessary, kanamycin resistance cassettes were excised by the Flp recombinase as previously described (81). Unless indicated, strains were grown in LB medium at 37°C either in liquid cultures with aeration or on solid medium containing 1.5% agar (80). When necessary, growth media were supplemented with ampicillin (25 or 125 μg/ml), l-arabinose (0.2%, wt/vol), bacitracin (100 μg/ml), chloramphenicol (20 μg/ml), erythromycin (25 μg/ml), vancomycin (25 or 75 μg/ml), kanamycin (25 μg/ml), tetracycline (25 μg/ml), MgCl_2_ (1 mM), EDTA (0.5 mM), oleic acid (10 mg/ml). MacConkey agar was commercially available from BD (catalog no. BD281810). Except for YhdP depletion strains, to monitor growth of strains, overnight cultures were diluted 1:1,000 in LB with the appropriate additives. Diluted cultures were delivered (200 μl) into a 96-well plate that was incubated at 37°C with continuous double orbital shaking in an Epoch2 BioTek reader. Growth was monitored every 30 min by measuring absorbance at 600 nm (OD_600_).

10.1128/mBio.02714-21.8 TABLE S3 Strains (A) and primers (B) used in this study. Download Table S3, XLSX file, 0.02 MB. Copyright © 2021 Ruiz et al. 2021 Ruiz et al. https://creativecommons.org/licenses/by/4.0/ This content is distributed under the terms of the Creative Commons Attribution 4.0 International license.

### YhdP depletion.

A YhdP depletion strain was constructed by recombineering as previously described (51). Briefly, primers 5YhdP_Pbad and YhdP_Pbad (Table S3B) were used to amplify the *bla*-*araC*-P_BAD_ region of pKD46 (82). The PCR product was used for recombineering to insert *bla*-*araC*-P_BAD_ between the −1 and +1 positions of the *yhdP* gene in the chromosome of the recombineering strain DY378 (83). Recombinants were selected at 30°C on medium containing 25 μg/ml ampicillin and confirmed by PCR analysis. The *yhdP* Ω(−1:: *bla araC*-P_BAD_) allele was introduced into various strains by using P1_vir_ transduction (80) by selecting ampicillin-resistant transductants. To deplete YhdP, cells grown overnight in LB with arabinose were washed once in LB and diluted 1:5,000 (vol/vol) in 25 ml LB with arabinose (+YhdP) or fucose (YhdP depletion) and grown at 37°C with shaking. Growth was monitored by measuring the OD_600_. For depletion on solid medium, overnight cultures grown in the presence of arabinose were serially diluted 1:10 in LB and stamped with a 48-pin replicator on the appropriate plates containing or lacking arabinose.

### Efficiency of plating assay.

Cultures grown overnight at 37°C in LB were serially diluted 1:10 (vol/vol). Dilutions were transferred with a 48-pin replicator to various plates and incubated overnight at 37°C. Efficiency of plating values was calculated by dividing the highest dilution with growth for each strain under each condition by the highest dilution with growth for that the wild-type strain MG1655 on LB agar.

### EDTA sensitivity assay.

Cultures grown overnight at 37°C in LB were diluted 1:1,000 (vol/vol) in LB and delivered to 96-well plates. EDTA was added and serially diluted to generate a 1:2 range of concentrations. After overnight incubation at 37°C, the MIC was determined as the lowest concentration of EDTA that inhibited growth as determined by OD_600_.

### Microscopy.

Cells grown in liquid media as indicated in each experiment were layered on a 1% agarose pad in LB and imaged using phase-contrast with a 100× oil immersion lens objective and a Nikon Eclipse Ti-E microscope equipped with a Nikon DS-QI1 cooled digital camera. See the supplemental material for details about cell measurements.

### Suppressor selection and mapping.

MacConkey-resistant suppressors were selected by plating 1 to 2 ml of overnight cultures of NR5161 on MacConkey agar plates. Selection plates were incubated overnight at 37°C. The *ydbH* suppressor alleles were mapped by P1_vir_ cotransduction frequency to tetracycline-resistant mini-Tn markers as described previously (84). Suppressor mutations were identified by amplifying the chromosomal *ydbH* locus via PCR and sequencing the resulting PCR product. A linked *tet2-3* mini-Tn insertion [IGR(*ynaE-uspF*)::*tet*] was used to move *ydbH* suppressor alleles into various strains via P1_vir_ transduction and demonstrate that they are solely responsible for suppression.

10.1128/mBio.02714-21.9 TEXT S1 Supplemental materials and methods, figure legends, and references. Download Text S1, DOCX file, 0.06 MB. Copyright © 2021 Ruiz et al. 2021 Ruiz et al. https://creativecommons.org/licenses/by/4.0/ This content is distributed under the terms of the Creative Commons Attribution 4.0 International license.
