# Supplementary material for: YhdP, TamB, and YdbH Are Redundant but Essential for Growth and Lipid Homeostasis of the Gram-Negative Outer Membrane
Source: mBio. 2021 Nov 16;12(6):e02714-21. doi: 10.1128/mBio.02714-21 (PMC8593681; doi:10.1128/mBio.02714-21)

A

No	Hit	Prob	E-value	P-value	Score	SS	Cols	Query HMM	Template HMM	
1	YP_026208.1 DUF3971-AsmA2 doma	99.6	5.3E-13	6.4E-18	112.4	21.2	144	1-145	30-174 (1266)	YhdP
2	NP_416568.1 suppressor of OmpF	99.4	1.6E-11	2E-16	96.5	14.7	124	5-130	29-163 (617)	AsmA
3	NP_417981.2 putative inner mem	99.4	1.5E-11	1.9E-16	98.0	12.5	125	4-130	31-179 (686)	YhjG
4	NP_418112.1 putative inner mem	99.1	1.3E-08	1.6E-13	79.8	14.3	122	1-144	25-148 (569)	YicH
5	NP_418642.1 translocation and	98.7	6.2E-07	7.5E-12	76.8	12.6	117	5-128	29-149 (1259)	TamB
6	NP_012064.1 Mdm31p [Saccharomy	97.7	0.0034	4.2E-08	50.2	12.5	135	5-143	145-322 (579)	Mdm31
7	NP_415899.1 putative membrane-	97.6	0.00091	1.1E-08	56.2	9.5	131	5-145	28-177 (879)	YdbH
8	NP_014790.4 Mdm32p [Saccharomy	96.9	0.066	8E-07	43.7	11.5	132	10-143	171-318 (622)	Mdm32
9	6A9J_A Endolysin,Autophagy-rel	81.7	13	0.00016	28.9	6.4	83	7-95	172-265 (389)	Atg2
10	NP_013060.1 membrane morphogen	77.5	1.3E+02	0.0016	31.0	17.0	141	3-151	1-200 (3144)	Vps13
11	6CBC_A Putative vacuolar prote	62.8	98	0.0012	23.5	12.4	135	7-149	6-190 (336)	Vps13

B

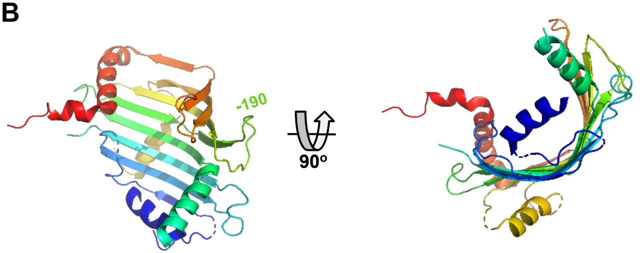

C

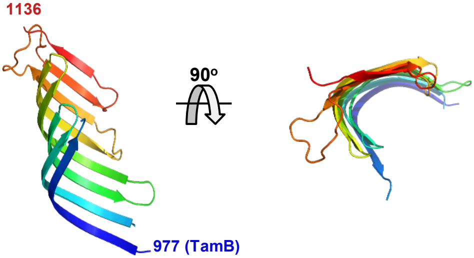

D

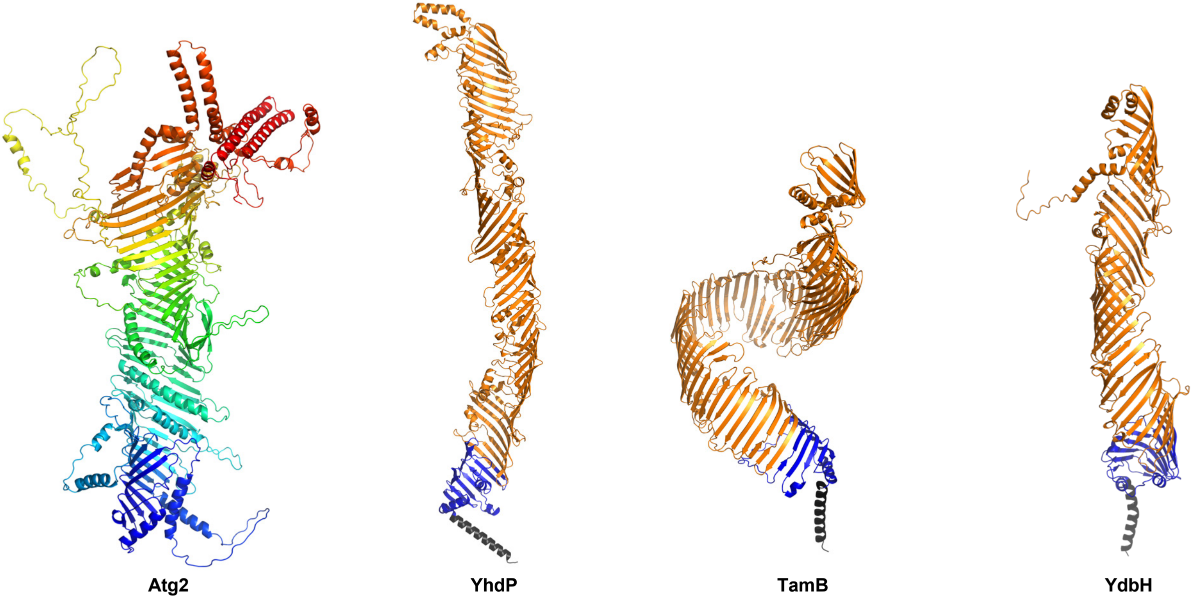

Supplement: FIG S1 [file mbio.02714-21-sf001.pdf]
