# Supplementary material for: YhdP, TamB, and YdbH Are Redundant but Essential for Growth and Lipid Homeostasis of the Gram-Negative Outer Membrane
Source: mBio. 2021 Nov 16;12(6):e02714-21. doi: 10.1128/mBio.02714-21 (PMC8593681; doi:10.1128/mBio.02714-21)
Supplement: FIG S2 [file mbio.02714-21-sf002.pdf]

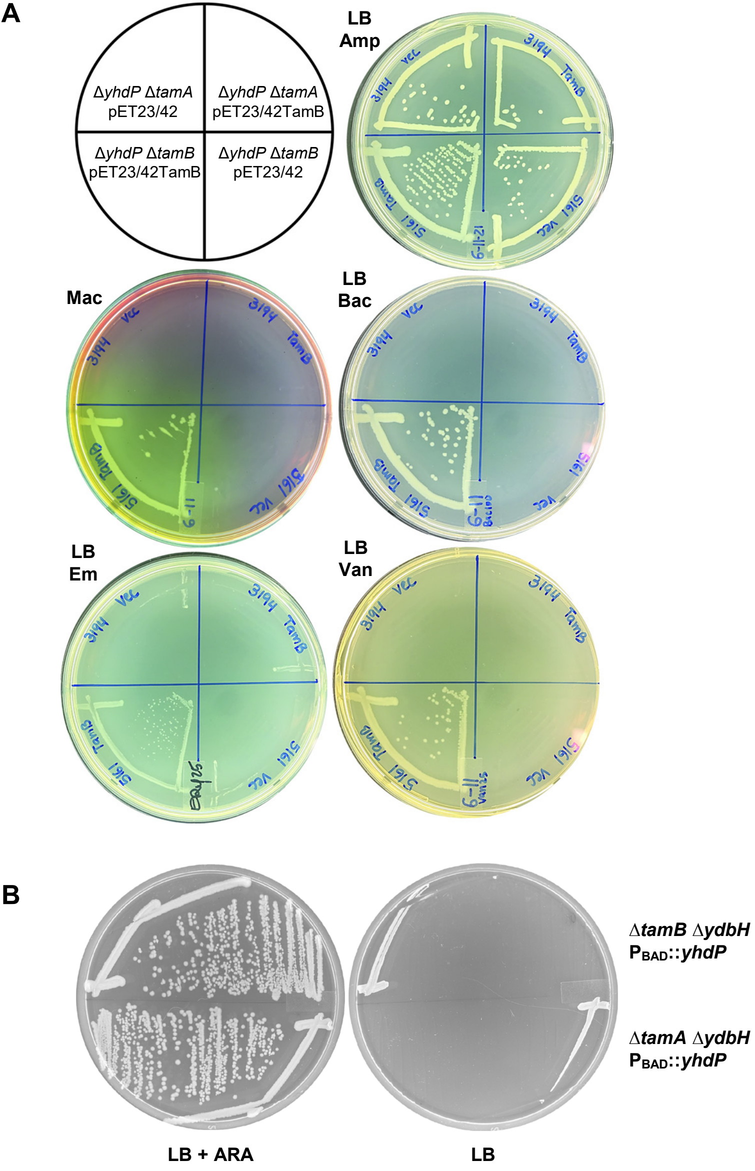
A
ΔyhdP ΔtamA pET23/42
ΔyhdP ΔtamA pET23/42TamB
ΔyhdP ΔtamB pET23/42TamB
ΔyhdP ΔtamB pET23/42
LB Amp
Mac
LB Bac
LB Em
LB Van
B
ΔtamB ΔydbH PBAD::yhdP
ΔtamA ΔydbH PBAD::yhdP
LB + ARA
LB
