# Supplementary figures and images for: YhdP, TamB, and YdbH Are Redundant but Essential for Growth and Lipid Homeostasis of the Gram-Negative Outer Membrane
Source: mBio. 2021 Nov 16;12(6):e02714-21. doi: 10.1128/mBio.02714-21 (PMC8593681; doi:10.1128/mBio.02714-21)

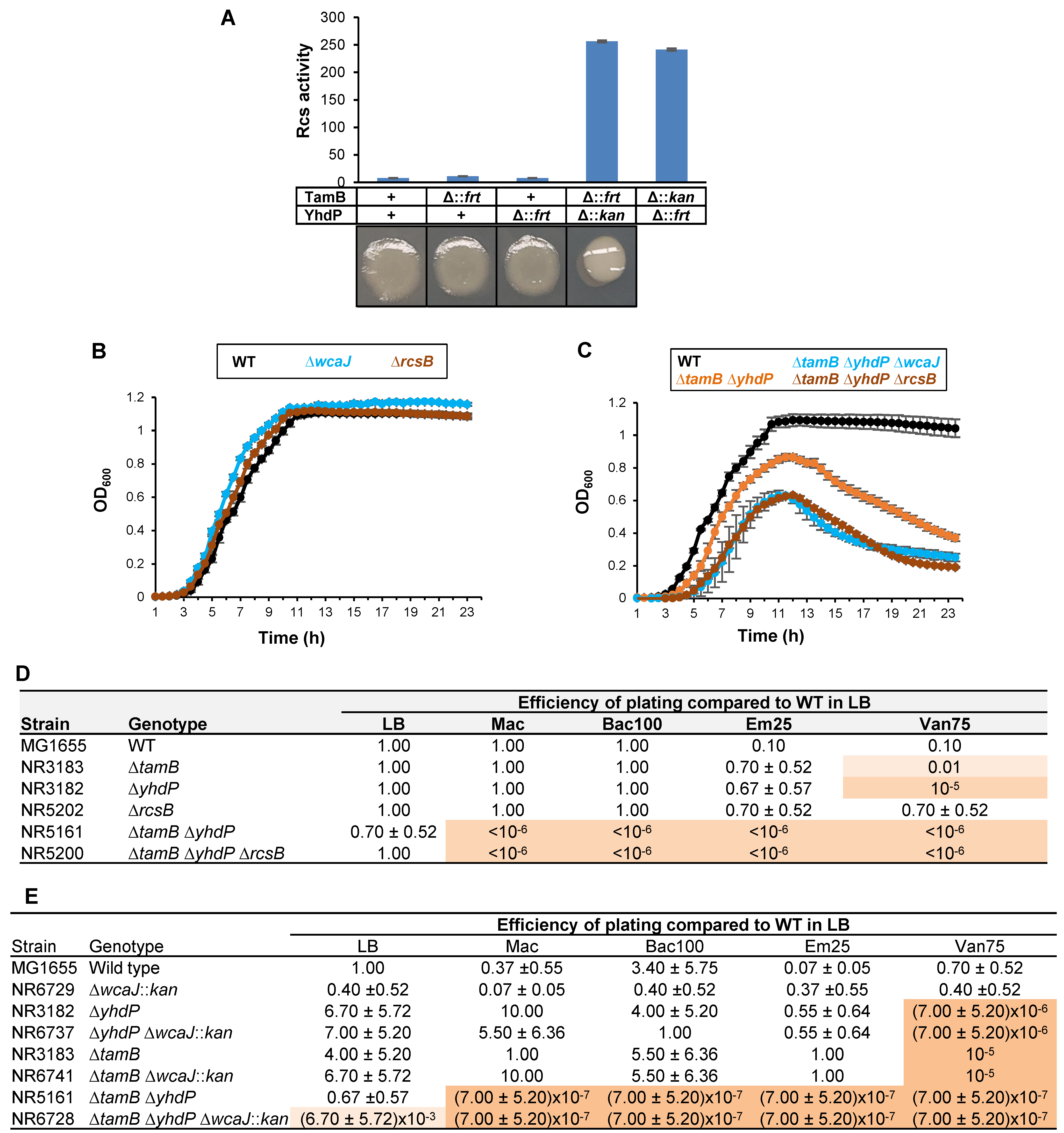

Supplement: FIG S3 [file mbio.02714-21-sf003.tif]

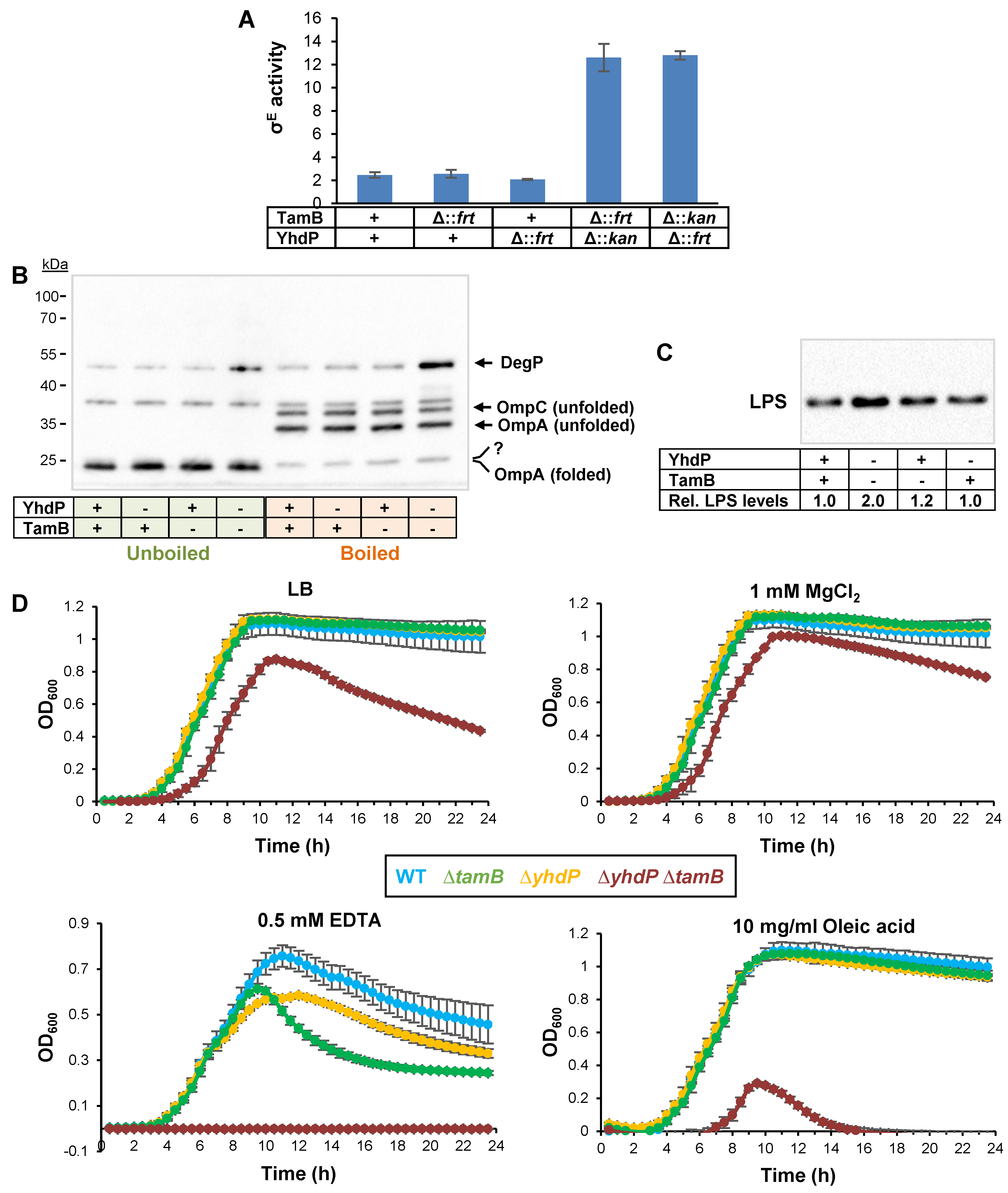

Supplement: FIG S4 [file mbio.02714-21-sf004.tif]

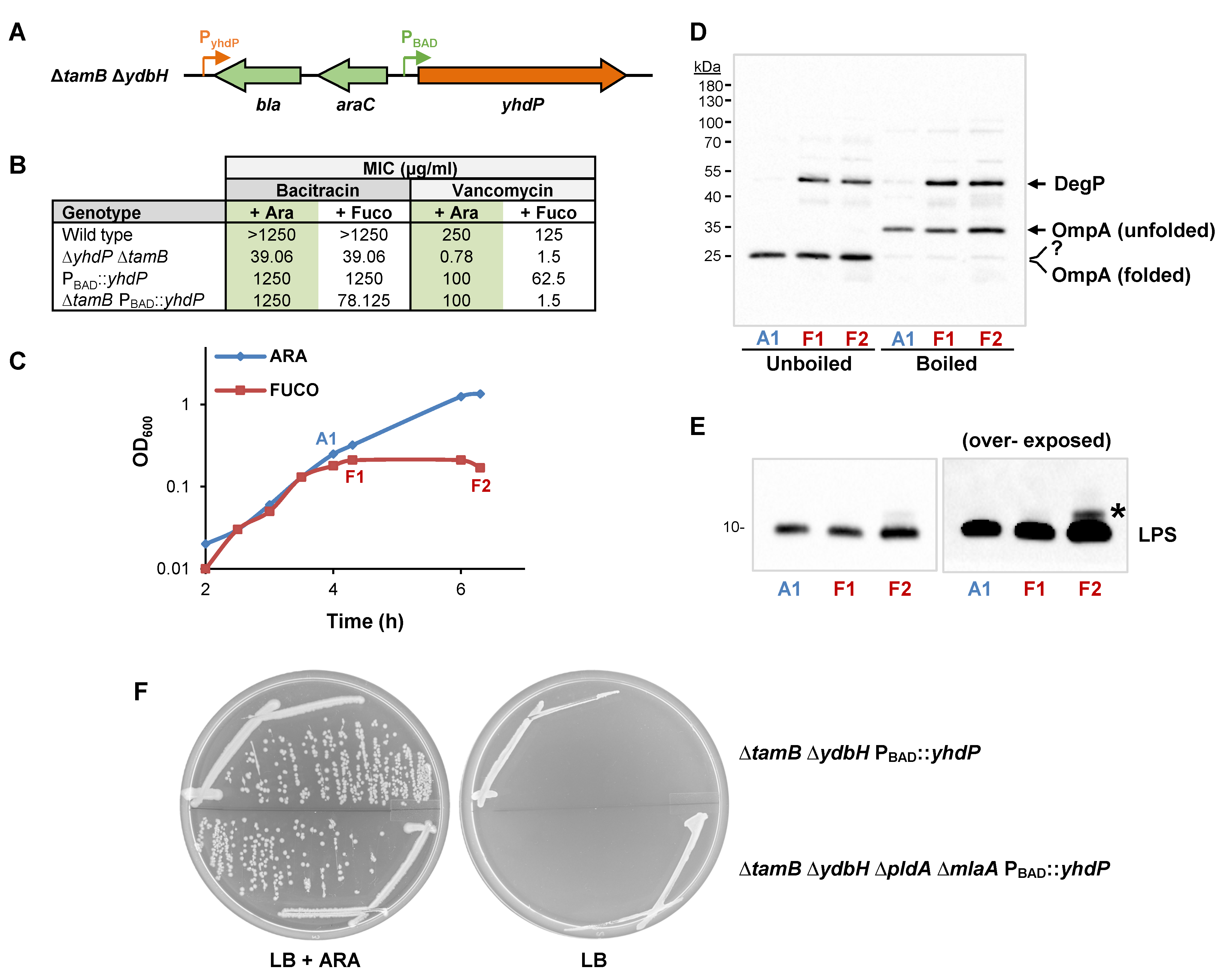

Supplement: FIG S5 [file mbio.02714-21-sf005.tif]
