# Supplementary material for: YhdP, TamB, and YdbH Are Redundant but Essential for Growth and Lipid Homeostasis of the Gram-Negative Outer Membrane
Source: mBio. 2021 Nov 16;12(6):e02714-21. doi: 10.1128/mBio.02714-21 (PMC8593681; doi:10.1128/mBio.02714-21)
Supplement: TABLE S2 [file mbio.02714-21-st002.pdf]

Table S2. Conservation of TamB and YhdP homologs in endosymbionts does not correlate with LPS production.

Endosymbiont	Genome accession	Total proteins	TamB homolog	YhdP homolog	LPS producer?[a]
Wigglesworthia glossinidia	NC_016893.1	634	WP_014354055.1		Yes
Baumannia cicadellinicola str. Hc	CP000238.1	595	ABF14103.1		No
Endosymbiont of Ctenarytaina eucalypti	CP003546.1	719	AFP85202.1		No
Vesicomyosocius okutanii HA	CF_000010405.1	948		WP_148178872.1	Yes
Buchnera aphidicola sp. APS	BA000003.2	574	BAB12807.1		No
Buchnera aphidicola str. Bp	AE016826.1	507	Q89AY7.1		No
Blochmannia floridanus	GCA_000043285.1	583	CAD83613.1		Yes

[a]The presence or absence of LPS was determined by searching for *lpx* (LPS synthesis) and *lpt* (LPS transport) genes in their respective annotated genomes.
