# Supplementary material for: YhdP, TamB, and YdbH Are Redundant but Essential for Growth and Lipid Homeostasis of the Gram-Negative Outer Membrane
Source: mBio. 2021 Nov 16;12(6):e02714-21. doi: 10.1128/mBio.02714-21 (PMC8593681; doi:10.1128/mBio.02714-21)
Supplement: TEXT S1 [file mbio.02714-21-s0001.docx]

Supplemental Materials and Methods

**Plasmid construction**

To construct pET23/42TamB, *tamB* was amplified from genomic DNA of MG1655 using primers TamB_BamHI_FP and TamB_XhoI_RP (Table S3B). To construct pET23/42YdbH/YnbE/YdbL, the *ydbH-ynbE-ydbL* locus was amplified from genomic DNA of MG1655 using primers YdbH-YdbL_BamH1_FP and YdbH-YdbL_XhoI_RP (Table S3B). The resulting PCR products were independently digested with BamHI and XhoI (restriction enzyme sites present in primers) and ligated by T4 DNA ligase into pET23/42 plasmid (1) that had been digested with the same restriction endonucleases. Proper construction of both plasmids was confirmed by DNA sequencing.

**Assay for protein release into growth medium**

Cultures grown overnight at 37°C in LB were pelleted by centrifugation for 2 min at 16,000 x g. Supernatants were collected and filtered through a 0.22 μm filter. For each strain, a 400-μL sample of cell-free supernatant was dried using a speed-vacuum concentrator vacuum at 60^o^C. Concentrates were mixed with 2x Laemmli sample solution, boiled for 10 min, and subjected to 10% SDS-polyacrylamide gel electrophoresis. Gels were stained with Blue-BANDit protein stain (Amresco), imaged with ChemiCoc XRS+ system, and visualized with ImageLab 5.2.1 software (Bio-Rad).

**Cell length and width measurements**

As described in Materials and Methods, cells grown in liquid media were layered on a 1% agarose pad with LB and imaged using phase-contrast with a 100× oil immersion objective lens and a Nikon Eclipse Ti-E microscope equipped with a Nikon DS-QI1 cooled digital camera. To measure cell length and width, we analyzed images with ObjectJ (2). To capture cells at a similar stage in the cell cycle, we measured cell length and width of each daughter of cells undergoing constriction during division (i. e. cells finalizing cell division with shape of the number 8) (3).

**Monitoring envelope stress responses with reporter fusions**

To monitor Rcs activity, the P*rprA*'-*lacZ*^+^ transcriptional reporter fusion from DH300 was used (4). To monitor σ^E^ activity, the *PrpoH3'-lacZ^+^* transcriptional reporter fusion from NR669 was used (5). Both transcriptional fusions were introduced into MG1655 *∆lacZYA::frt* by genetic transduction with λ lysogens obtained from DH300 and NR669 as previously described (6). These strains were then used to make Δ*yhdP* and Δ*tamB* derivatives. For the reporter assays, cells carrying reporter fusions were grown to OD_600_ ~0.5. A 1-mL sample was collected from each culture, and cells pelleted by centrifugation (16,000 x g, 1 min) and resuspended in 1 mL of Z-buffer (0.06 M Na_2_HPO_4_.7H_2_O, 0.04 M NaH_2_PO_4_.H_2_O, 0.01 M KCl, 0.001 M MgSO_4_.7H_2_O, 0.05 M H 7.0, 0.05 M β-mercaptoethanol, pH adjusted to 7.0). Two drops of chloroform and one drop of 0.1% SDS were added to each sample using a transfer pipette. Samples were vortexed for 10 s. Permeabilized cells were transferred to a 96-well plate, mixed with 100 µl of 5 mg/ml ONPG in Z-Buffer, and their absorbance at 420 nm every 10 s for 10 min in an xMark reader. To calculate relative levels of β-galactosidase activity and normalize samples by their cell density, the rate value (mOD_420_/time) obtained from each sample was divided by the OD_600_ value of its respective culture.

**LPS levels**

Cells from a 200-μL sample of cultures grown to OD_600_ ~0.5-0.6 were collected by centrifugation (16,000 x g, 2 min) and resuspended in a volume of Laemmli sample solution equivalent to OD_600_/10 in μL. A 5-μL portion of each sample was loaded onto 15% SDS-polyacrylamide gels. After electrophoresis, LPS was transferred onto polyvinylidene difluoride (PVDF) membranes for 30 min at 10 V in a semi-dry transfer cell (BioRad). PVDF membranes were probed with mouse anti-LPS (1:10,000 dilution; Bio-Rad 4329-5004) followed by anti-mouse horseradish peroxidase-conjugated secondary antibody (1:10,000 GE Healthcare Life Sciences). Signal was developed using Clarity Western ECL substrate (Bio-Rad), imaged with Chemidoc XRS+ system, and visualized with ImageLab 5.2.1 software (Bio-Rad). Images were captured before signal was saturated.

**OMP and DegP levels**

Cells from a 1-mL sample of cultures grown to OD_600_ 0.6 were collected by centrifugation (16,000 x g, 2 min). and resuspended in 50 μL of BugBuster Protein Extraction Reagent (Novagen). After shaking for 20 min at room temperature, samples were mixed with 50 μL of 2x Laemmli sample solution and divided into two tubes that were either boiled or not for 10 min. Equal volumes from boiled and not boiled samples were loaded onto 10% SDS-polyacrylamide gels. After electrophoresis, proteins were transferred onto PVDF membranes for 1 h at 10 V in a semi-dry transfer cell (BioRad). Membranes were probed with rabbit antiserum raised against DegP, LamB (which also recognizes OmpA) or OmpC, (1:25,000 dilution) followed by anti-rabbit horseradish peroxidase-conjugated secondary antibody (1:10,000 GE Healthcare Life Sciences). Signal was developed using Clarity Western ECL substrate (Bio-Rad), imaged with Chemidoc XRS+ system, and visualized with ImageLab 5.2.1 software (Bio-Rad).

**AlphaFold structure predictions**

Model structures for Atg2 (UniProt 53855), YhdP (UniProt P46474), TamB (UniProt P 39321), YdbH (UniProt P52645), were obtained from the AlphaFold Protein Structure Database using the https://alphafold.ebi.ac.uk/ server (7).

**Supplemental Material References**

1. Wu T, McCandlish AC, Gronenberg LS, Chng SS, Silhavy TJ, Kahne D. 2006. Identification of a protein complex that assembles lipopolysaccharide in the outer membrane of *Escherichia coli*. Proc Natl Acad Sci U S A 103:11754-9.

2. Vischer NO, Verheul J, Postma M, van den Berg van Saparoea B, Galli E, Natale P, Gerdes K, Luirink J, Vollmer W, Vicente M, den Blaauwen T. 2015. Cell age dependent concentration of *Escherichia coli* divisome proteins analyzed with ImageJ and ObjectJ. Front Microbiol 6:586.

3. Yao Z, Davis RM, Kishony R, Kahne D, Ruiz N. 2012. Regulation of cell size in response to nutrient availability by fatty acid biosynthesis in *Escherichia coli*. Proceedings of the National Academy of Sciences of the United States of America 109:E2561-8.

4. Majdalani N, Hernandez D, Gottesman S. 2002. Regulation and mode of action of the second small RNA activator of RpoS translation, RprA. Mol Microbiol 46:813-26.

5. Button JE, Silhavy TJ, Ruiz N. 2007. A suppressor of cell death caused by the loss of sigmaE downregulates extracytoplasmic stress responses and outer membrane vesicle production in *Escherichia coli*. Journal of bacteriology 189:1523-30.

6. Silhavy TJ, Berman ML, Enquist LW. 1984. Experiments with gene fusions. Cold Spring Harbor Laboratory, Cold Spring Harbor, NY.

7. Jumper J, Evans R, Pritzel A, Green T, Figurnov M, Ronneberger O, Tunyasuvunakool K, Bates R, Zidek A, Potapenko A, Bridgland A, Meyer C, Kohl SAA, Ballard AJ, Cowie A, Romera-Paredes B, Nikolov S, Jain R, Adler J, Back T, Petersen S, Reiman D, Clancy E, Zielinski M, Steinegger M, Pacholska M, Berghammer T, Bodenstein S, Silver D, Vinyals O, Senior AW, Kavukcuoglu K, Kohli P, Hassabis D. 2021. Highly accurate protein structure prediction with AlphaFold. Nature 596:583-589.

8. Gabler F, Nam SZ, Till S, Mirdita M, Steinegger M, Soding J, Lupas AN, Alva V. 2020. Protein sequence analysis using the MPI Bioinformatics Toolkit. Curr Protoc Bioinformatics 72:e108.

9. Kumar N, Leonzino M, Hancock-Cerutti W, Horenkamp FA, Li P, Lees JA, Wheeler H, Reinisch KM, De Camilli P. 2018. VPS13A and VPS13C are lipid transport proteins differentially localized at ER contact sites. J Cell Biol 217:3625-3639.

10. Osawa T, Kotani T, Kawaoka T, Hirata E, Suzuki K, Nakatogawa H, Ohsumi Y, Noda NN. 2019. Atg2 mediates direct lipid transfer between membranes for autophagosome formation. Nat Struct Mol Biol 26:281-288.

11. Otomo T, Maeda S. 2019. ATG2A transfers lipids between membranes *in vitro*. Autophagy 15:2031-2032.

12. Valverde DP, Yu S, Boggavarapu V, Kumar N, Lees JA, Walz T, Reinisch KM, Melia TJ. 2019. ATG2 transports lipids to promote autophagosome biogenesis. J Cell Biol 218:1787-1798.

13. Dimmer KS, Jakobs S, Vogel F, Altmann K, Westermann B. 2005. Mdm31 and Mdm32 are inner membrane proteins required for maintenance of mitochondrial shape and stability of mitochondrial DNA nucleoids in yeast. J Cell Biol 168:103-15.

14. Meredith TC, Mamat U, Kaczynski Z, Lindner B, Holst O, Woodard RW. 2007. Modification of lipopolysaccharide with colanic acid (M-antigen) repeats in *Escherichia coli*. J Biol Chem 282:7790-8.

15. Ruiz N, Gronenberg LS, Kahne D, Silhavy TJ. 2008. Identification of two inner-membrane proteins required for the transport of lipopolysaccharide to the outer membrane of *Escherichia coli*. Proceedings of the National Academy of Sciences of the United States of America 105:5537-42.

**Supplemental Figure Legends**

Fig. S1. The N-terminal periplasmic region of YhdP and AsmA-like paralogs is homologous to chorein-N domains present in eukaryotic lipid transporters. Hit list produced by searching residues 30-180 of YhdP with HHpred [Supplemental Material Text reference (8)] showing that the N-terminal periplasmic region of the six *E. coli* AsmA-like proteins (in blue) share sequence homology with each other and the N terminus of members of the eukaryotic chorein_N family of lipid transporters Atg2 and Vps13 (in orange), which are involved in inter-organelle lipid transport [Supplemental Material Text reference (9-12)]. Mdm31 and Mdm32 (in green) are also eukaryotic proteins that have been implicated in inter-membrane transport between the mitochondrial inner and outer membranes [Supplemental Material Text reference (13)]. The sequence [referred to as YhdP(30-180)] corresponding to the first 151 residues (positions 30-180) of the predicted periplasmic region of YhdP was used to search for sequence homology in the PDB_mmCIF30_13_Sept and NCBI_Conserved_Domains(CD)_v3.18 databases. The top 11 of 15 hits are shown (hits 12-15 had <50% probability score). Prob refers to the probability of being a true positive hit. The E‐value is the average number of false positives with a score better than the one obtained for YhdP(30-180), while the P‐value is the E‐value divided by the number of sequences in the database. B) Cartoon diagram of the crystal structure (PDB 6CBC) of residues 6-320 of the fungal *Chaetomium thermophilum* Vps13 protein containing the chorein-N domain. HHpred predicts homology with AsmA-like proteins up to *ca.* residue 190 (labeled). C) Cartoon diagram of the crystal structure (PDB 5VTG) of the β-taco fold composed of residues 977-1136 of TamB from *E. coli*. Crystal structures are colored from blue (N terminus) to red (C terminus). Structure pictures were generated using PyMOL Molecular Graphics System (Schrödinger, LLC). D) Cartoon representation of the model structures for Atg2 (UniProt 53855), YhdP (UniProt P46474), TamB (UniProt P 39321), YdbH (UniProt P52645) generated by AlfaFold (7). The Atg2 chain is colored using the rainbow pattern from blue (N-terminus) to red (C-terminus). In YhdP, TamB, and YdbH, the predicted transmembrane helix is in black, the chorein-N domain in blue, and the AsmA-like domain in orange.

Fig. S2. TamA is required for TamB function. A) Strains NR5161 (∆*tamB* ∆*yhdP*) and NR3194 (∆*tamA* ∆*yhdP*) are sensitive to bile salts and antibiotics, and indistinguishable under all conditions we tested. TamB encoded in plasmid pET23/42TamB restores resistance to bile salts and antibiotics when introduced into NR5161 but not NR3194. The pET23/42 control plasmid does not complement either strain. MacConkey (Mac) plates or LB plates containing 125 μg/ml ampicillin (resistance conferred by plasmids), 100 μg/ml bacitracin (Bac), 25 μg/ml erythromycin (Em), or 25 μg/ml vancomycin (Van) were photographed after overnight incubation at 37^o^C. B) TamA is required for viability in the absence of YdbH and YhdP. Replacing the ∆*tamB*::*frt* allele of NR6834 (MG1655 ∆*tamB*::*frt* ∆*ydbH*::*frt tet2-3* *yhdP*Ω-1::*bla* *araC* P_BAD_) with ∆*tamA*::*kan* resulted in strain NR7140 (MG1655 ∆*tamA*::*kan* ∆*ydbH*::*frt tet2-3* *yhdP*Ω-1::*bla* *araC* P_BAD_), which is dependent on arabinose for growth like its parental strain NR6834. LB plates with and without arabinose (ARA) were streaked with NR6834 and NR7140 and incubated overnight at 37^o^C

Fig. S3. Loss of colanic acid capsule impairs growth of the ∆*yhdP* ∆*tamB* mutant. A) The ∆*tamB* ∆*yhdP* mutant, unlike its single mutant parents and the wild-type strain, is mucoid (photos of colonies bellow graph). In agreement, the Rcs envelope stress response is upregulated in the ∆*tamB* ∆*yhdP* double mutant. We constructed derivatives of a strain [MG1655 ∆lacZYA::FRT λimm 21 φ (P*rprA*'-*lacZ*^+^)] carrying a transcriptional *lacZ* fusion to the *rprA* promoter, which is upregulated by Rcs. Relative LacZ levels (normalized with respect to optical density) in various strains grown exponentially in LB and 37^o^C. Data represent the average and standard deviation of three biological replicates. B) The *wcaJ* gene is required for the synthesis of colanic acid capsule and its expression is up-regulated by RcsB, so the loss of either *wcaJ* or *rcsB* abolished mucoidy in the ∆*tamB* ∆*yhdP* double mutant. Introducing either a ∆*wcaJ* or a ∆*rcsB* null allele into the wild-type strain (WT) did not affect growth in LB and 37^o^C, as monitored by OD_600_. C) Growth in LB and 37^o^C was determined by OD_600_ for strains MG1655 (wild type), NR5161 (∆*tamB* ∆*yhdP*), NR6728 (∆*tamB* ∆*yhdP* ∆*wcaJ*), and NR5200 (∆*tamB* ∆*yhdP* ∆*rcsB).* The loss of either *wcaJ* or *rcsB* similarly compromises the growth of cells lacking TamB and YhdP. D and E) Deletion of *rcsB* (D) or *wcaJ* (E) did not change the OM permeability of the wild-type or the ∆*tamB* ∆*yhdP* strains. Data represent the average and standard deviation of three biological replicates. If not shown, standard deviation equals zero.

Fig. S4. Loss of both TamB and YhdP activates the σ^E^ stress response and increases LPS levels. A) The σ^E^ envelope stress response is induced in the *tamB* ∆*yhdP* double mutant, unlike in its ancestor strains derivatives of a strain [MG1655 ∆lacZYA::FRT λRS45 φ (P*rpoH3*'-*lacZ*^+^)] carrying a transcriptional *lacZ* fusion to the *rpoH3* promoter, which is upregulated by σ^E^. Relative LacZ levels (normalized with respect to OD_600_) in various strains grown exponentially in LB and 37^o^C. Data represent the average and standard deviation of three biological replicates. B) Whole-cell protein extracts obtained with BugBuster from exponentially growing cells (OD_600_ ~0.6 LB, 37^o^C) were subjected to electrophoresis and immunoblotting for the periplasmic protease DegP (which is up-regulated by the σ^E^ envelope stress response) and the β-barrel outer membrane proteins OmpA and OmpC. In unboiled samples, OmpA migrates in its folded conformation (~25 kDa) and OmpC is not recognized by the antiserum. Boiling samples denatures and unfolds OmpA and OmpC, which migrate as indicated. An unidentified band above folded OmpA is marked with a question mark. The loss of YhdP and TamB increases the levels of DegP but has no detectable effect on the folding of OmpA and OmpC. C) Samples in panel C were also subjected to electrophoresis and immunoblotting to compare levels of LPS. The loss of YhdP and TamB increases the levels of LPS. Intensity of the signal in the LPS band was measured and values shown below the immunoblot represent relative values across samples that were calculated by setting levels in the wild-type strain MG1655 to 1.0. Immunoblots shown in panels B and C are representative of at least three independent experiments. D) Effect of various additives to the growth of strains lacking TamB and/or YhdP. Growth curve of wild-type strain MG1655 (WT) and derivatives carrying ∆*tamB* and/or ∆*yhdP* alleles in LB at 37^o^C. When indicated above the graph, MgCl_2_ (to stabilize LPS on the cell surface), EDTA (to extract LPS from the cell surface), or the fatty acid oleic acid were added at the specified concentration at the beginning of the experiment. Data represent the average and standard deviation of three biological replicates.

Fig. S5. Phenotypic analysis of the ∆*tamB* ∆*ydbH* YhdP-depletion strain. A) Relevant genetic features of strain NR5921 (MG1655 ∆*tamB*::*frt* ∆*ydbH*::*kan* *yhdP*Ω-1::*bla* *araC* P_BAD_). B) Minimal inhibitory concentration (MIC) assay revealed that depletion (+Fuco) of YhdP in a ∆*tamB* mutant increases sensitivity to bacitracin and vancomycin with respect to YhdP-replete conditions (+Ara), similarly to that observed in a ∆*tamB* ∆*yhdP* mutant. C) Growth of YhdP-depletion strain NR5921 at 37^o^C in LB in the presence of arabinose (ARA) or fucose (FUCO) to induce or repress expression of *yhdP*, respectively. An overnight culture of NR5921 was grown in LB the presence of arabinose at 37^o^C. After a 1:5,000 dilution in LB containing either arabinose or fucose, growth at 37^o^C was measured by monitoring OD_600_. Depletion of YhdP in the ∆*tamB* ∆*ydbH* mutant arrests growth. Cells were collected at points labelled A1, F1 and F2 to prepare whole cell extracts for immunoblotting shown in panels D and E. D) Whole-cell protein extracts obtained with BugBuster (at times indicated in panel C) were subjected to electrophoresis and immunoblotting for the periplasmic protease DegP (which is up-regulated by the σ^E^ envelope stress response) and the β-barrel outer membrane protein OmpA. In unboiled samples, OmpA migrates in its folded conformation (~25 kDa), but boiling samples denatures and unfolds OmpA, which migrates more slowly as indicated. An unidentified band above folded OmpA is marked with a question mark. Depletion of YhdP in the ∆*tamB* ∆*ydbH* mutant increases the levels of DegP but has no detectable effect on the folding of OmpA. E) Samples in panel D were also subjected to electrophoresis and immunoblotting to compare levels of LPS. Depletion of YhdP increases the levels of LPS and leads to the appearance of a band of slightly higher mass (marked with asterisk) that likely results from LPS being modified with colanic acid because of up-regulation of colanic acid production and/or accumulation of LPS at the IM [Supplemental Material Text reference (14, 15)]. Data shown are representative of at least three independent experiments. F) The combined loss of PldA and MlaA does not suppress the essentiality of TamB, YhdP, and YdbH. Introducing the ∆*pldA* ∆*mlaA* alleles into the YhdP-depletion strain lacking TamB and YdbH, NR6834 (MG1655 ∆*tamB*::*frt* ∆*ydbH*::*frt tet2-3* *yhdP*Ω-1::*bla* *araC* P_BAD_), does not suppress dependence on the inducer arabinose for growth. LB plates with and without arabinose (ARA) were streaked with NR6834 and NR7030 (MG1655 ∆*tamB*::*frt* ∆*ydbH*::*frt* ∆*pldA*::*frt* ∆*mlaA*::*kan* *tet2-3* *yhdP*Ω-1::*bla* *araC* P_BAD_) and incubated overnight at 37^o^C.
